# Incorporation of a Natural Deep Eutectic Solvent-Based System as a Cryoprotectant in Solid Lipid Nanoparticles: Advancing toward Industrial Scalability

**DOI:** 10.1021/acsnanoscienceau.5c00097

**Published:** 2025-10-01

**Authors:** Isadora Florêncio, Marina M. Simões, Karen L. R. Paiva, Luane de Almeida Salgado, Ariane P. Silveira, Tathyana B. Piau, Cesar K. Grisolia, Victor Carlos Mello, Sônia N. Báo

**Affiliations:** † Laboratory of Microscopy and Microanalysis, Department of Cell Biology, Institute of Biological Sciences, 28127 University of Brasília, Brasília-DF 70910-900, Brazil; ‡ Laboratory of Nanobiotechnology, Department of Genetics and Morphology, Institute of Biological Sciences, University of Brasilia, Brasília-DF 70910-900, Brazil; § Cooil Cosmetics, Brasília-DF 72622-401, Brazil; ∥ Laboratory of Genetic Toxicology, Department of Genetics and Morphology, Institute of Biological Sciences, 28127 University of Brasília, Brasília-DF 70910-900, Brazil

**Keywords:** particle concentration, freezing, sustainable nanotechnology, industrial scale-up, glycerol, choline chloride, citric acid

## Abstract

The industrial-scale production of solid lipid nanoparticles (SLNs) faces significant challenges, such as degradation during freezing and the reliance on toxic solvents for cryoprotection and active ingredient solubility, hindering their effective storage and commercialization. This study presents an innovative and sustainable approach to SLNs formulation by incorporating a natural deep eutectic solvent-based system (NaDES) as an integral part of the system. The research evaluated the cryoprotective potential of NaDES at different concentrations and freezing methods, demonstrating their ability to stabilize aluminum chloride phthalocyanine (AlClPc)-loaded SLNs during freezing and enable drying protocols to enhance particle concentration. SLNs formulations with high colloidal stability were obtained, containing 12.5% NaDES, based on low-energy methodologies and high added value. Additionally, the use of NaDES enabled a 12.5% reduction in the water content in the formulation and acted as an efficient cryoprotectant, allowing for the freezing of SLNs without compromising particle integrity. These advancements suggest a greener and potentially scalable methodology for SLNs production, positioning NaDESs as promising cryoprotectants that may enhance formulation stability, improve commercial viability, and reduce production and storage costs. This innovation may represent an initial step toward improving nanoparticle preservation and facilitating future industrial translation, with the potential to broaden nanoparticle application in nanotechnology.

## Graphical abstract

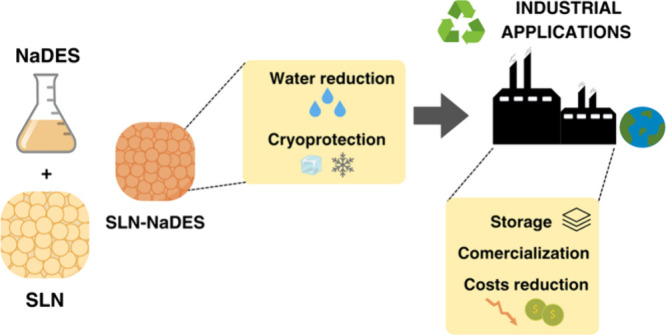

## 1 Introduction

The global economic landscape is no longer the same, and the relentless pursuit of technological innovations, driven by practical problem-solving and based on scalable ideas, has led to the emergence of companies focused on high-risk investments and major scientific discoveries.^1^ According to the Startup Genome Report (2023), in just the last five years, Deep Tech startup sectors have grown by 326%, standing out as highly successful business models.^2^

As a rich source of knowledge and infrastructure, the academic environment is often the place where many of these companies are created and developed. However, mainly due to the lack of funding and intrinsic challenges in the process, much of the developed technology fails in attempt to go beyond academia, being this one of the greatest obstacles to bridging the gap between scientific research and the market, especially when discussing nanotechnology, a field that has gained significant prominence among highly innovative technologies.^3^ However, despite the various advantages presented by nanostructured systems, nanoparticles still face challenges that hinder the progress of technologies from the laboratory bench to industry shelves, making industrial scaling harder. This step is essential for integrating the high-quality technologies that are part of highly successful Deep Tech startups. Regarding solid lipid nanoparticles (SLNs), this issue is even more common.^4^

SLNs emerged in the 1990s as a promising alternative to some existing nanocarriers such as polymeric nanoparticles, liposomes, and nanoemulsions. Among the advantages of SLNs are their low toxicity in biological systems, exhibiting high biocompatibility and biodegradability, as well as great physical stability provided by their lipid matrix, which allows adequate encapsulation of active ingredients.^5,6^ For these reasons, SLNs have become widely used as carrier systems for drug delivery, aiming at treating various diseases, such as cancer.^7^ When associated with aluminum chloride phthalocyanine (AlClPc), a well-studied photosensitizer for therapeutic applications, SLNs can be applied in a variety of contexts, including photodynamic therapy (PDT) for the treatment of tumors, dermatological and cosmeceutical therapies, and fluorescence-based imaging applications, among others.^8–10^ Despite the advantages of SLNs, their long-term storage is a challenge due to the colloidal instability inherent to the suspension. This instability may lead to the redistribution of lipid constituents, accompanied by particle aggregation, drug leakage, microbial contamination, and the oxidation of lipid components.^11–13^ Besides, SLN also presents issues related to susceptibility to various stress conditions, such as exposure to freezing, which promotes their degradation and prevents storage under low temperatures, complicating their widespread commercialization.^14^

Freezing represents a critical step in the preservation of colloidal systems, such as SLNs, particularly for long-term storage. This process contributes to the physicochemical stabilization of the formulation by significantly reducing water activity, thereby minimizing degradation reactions and colloidal instability.^15,16^ Moreover, freezing plays an essential preparatory role in lyophilization—a technique that involves the removal of water from previously frozen samples to concentrate the material and extend its shelf life. Particle concentration through lyophilization enables subsequent resuspension in reduced volumes of the diluent, allowing the preparation of more concentrated formulations. This strategy is particularly advantageous for applications requiring higher drug payloads, such as controlled release systems or the administration of potent therapeutics in low-volume doses.^17–19^ Thereby, technological alternatives aimed at promoting the feasibility of scaling these nanoparticles, enabling their freezing without degradation, and improving their storage and transport emerge as solutions to mitigate the obstacles faced by SLNs on the path to industrial scaling. Thus, techniques that allow for cryopreservation, as well as concentration of these particles without promoting degradation, could become the key to addressing the challenges associated with SLNs.

In the context of the present work, an aqueous solution system of organic compounds based on natural deep eutectic solvents (NaDESs) was investigated for its potential to address these limitations associated with SLNs, allowing for their cryopreservation and enabling methodologies to concentrate these particles. This approach may offer advantages for the scaling and commercialization of such technology in addition to representing an innovative association not yet described in the literature.

NaDESs comprise a class of green solvents formed by mixing organic components from the primary metabolism of living organisms, such as acids, sugars, choline derivatives, and amino acids. When mixed in specific molar ratios, these components create a homogeneous liquid mixture at room temperature with a melting point significantly lower than that of the individual components. The intermolecular interaction occurs through hydrogen bonding, where one component acts as a hydrogen donor and the other as an acceptor. This charge displacement lowers the melting point, characterizing it as a eutectic mixture, which enables NaDESs to dissolve many hard-to-dissolve substances, including those insoluble in water, such as aluminum chloride phthalocyanine (AlClPc), making them very interesting.^20^

Studies have already demonstrated the cryoprotective potential of NaDESs for biological systems, such as cells, in that these solvents act by preventing the formation of ice crystals or reducing this process.^21^ However, the capacity of NaDESs to act as cryoprotectants in nanocarriers, particularly those aimed at drug delivery, has not yet been demonstrated.

In this context, the present work aimed to develop a methodology to promote the cryopreservation and concentration of SLNs carrying AlClPc using an NaDES, enhancing the feasibility of SLN-related protocols for industrial applications.

## Materials and Methods

### Materials

Murumuru butter (*Astrocaryum murumuru*) was purchased from Amazon Oil (Ananindeua, Pará, Brazil). Osmium tetroxide was purchased from Electron Microscopy Science, USA. AlClPc, citric acid, Brij O10 (Polyoxyethylene (10) oleyl ether), choline chloride, ethanol, and glycerol were purchased from Sigma-Aldrich, USA.

### Preparation of the NaDES

The NaDES selected for the formulation of the SLNs developed in this study was based on the solvent previously developed and characterized by Silva et al.^22^ and consists of a mixture composed of choline chloride (C_5_H_14_ClNO), glycerol (C_3_H_8_O_3_), and citric acid (C_6_H_8_O7) in a molar ratio of 0.5:2:0.5. The reagents were weighed, and the mixture was stirred at 80 °C for 30 min until a homogeneous liquid was obtained. Subsequently, water was added in a ratio of 25% (v/v), following the protocol previously described by the authors.^22^

The selection of the solvent was grounded in the integration of physicochemical and molecular criteria, combined with its feasibility of application, in addressing the challenges investigated in the present study. NaDESs composed of choline chloride, glycerol, and citric acid are extensively described in the literature and are well established as model systems that accurately represent the compositional diversity of natural eutectic mixtures.^23,24^ Furthermore, these systems have gained increasing prominence in pharmaceutical and cosmetic applications, which, although still at an early stage, substantially expand their prospective spectrum for biological applicability.^25,26^ In addition, their straightforward preparation methodology, low energy demand, and broad availability of their constituent compounds are key aspects to be considered, particularly in the context of prospective studies aimed at scalability and technological translation.

Thus, given the previous context investigations of this solvent and its characteristics reported by Silva et al.,^22^ it emerged as an appropriate candidate for the methodological assessment of the cryoprotective role in SLNs formulations in this study.

### Preparation of SLNs

Four SLN formulations were developed with or without AlClPc. Two of these formulations do not contain NaDES (SLN-Blank and SLN-AlClPc), while the other two include NaDES in their composition (SLN-Blank-NaDES and SLN-AlClPc-NaDES), as detailed in Table 1. It is important to note that formulations designated as “Blank” do not contain AlClPc.

**Table 1:** Nanoparticles Developed and Their Respective Components

**SLN**	**water**	**murumuru butter**	surfactant (Brij O10)	**AlClPc**	**NaDES**
SLN-Blank	X	x	x		
SLN-Blank-NaDES	X	x	x		x
SLN-AlClPc	X	x	x	x	
SLN-AlClPc-NaDES	X	x	x	x	x

The SLNs were prepared using the phase inversion temperature (PIT) method, with formulations adapted from the methodology previously developed by Mello et al.^27^ The oily phase is composed of murumuru butter and surfactant (Brij O10) in a ratio of 2:1, respectively, which corresponds to 7.5% (w/v) of the formulation. Murumuru butter comes from Amazonian biodiversity, and for this reason, this study is registered in the National System for the Management of Genetic Heritage and Associated Traditional Knowledge (SisGen) under registration number A1563A6.

#### SLNs without NaDES-Based System (NaDES)

For the preparation of SLNs without NaDES (SLN-Blank and SLN-AlClPc), the oily phase composed of murumuru butter and the surfactant Brij O10 was previously heated to 65 °C until the components completely melted. Subsequently, the aqueous phase composed of 40 mL of distilled water at 65 °C was added, and the mixture was kept under constant stirring until 80 °C, when it was cooled under a constant flow of water until reaching 25 °C. For the SLN-AlClPc, after the melting of the oily phase, an ethanolic solution of AlClPc was added, with a final concentration of 20 μM in the formulation.

#### SLNs Containing NaDES-Based System (NaDES)

For the formulation of SLNs containing NaDES (SLN-Blank-NaDES and SLN-AlClPc-NaDES), the protocol was similar; however, in this preparation, NaDES was added to the formulation in the oily phase, immediately after the melting of murumuru butter and Brij O10. Following the addition of NaDES to the oily phase, an aqueous phase containing only water was added to the formulation. In the aqueous phase, the volume removed from water was proportional to the volume of NaDES added in the oily phase. This protocol was applied for all tested solvent concentrations (1.25, 2.5, 5, 7.5, 10, 12.5, 15, 20, and 25%). For SLNs-AlClPc-NaDES, after the melting of the oily phase, an ethanolic solution of AlClPc was added, with a final concentration of 20 μM in the formulation.

### Freezing of SLN

To assess the cryoprotective effect of NaDES containing choline chloride, glycerol, and citric acid (0.5:2:0.5) in SLN formulations, three types of freezing were tested: slow (−20 °C), rapid (−80 °C), and ultrafast (−196 °C). For each temperature, the colloidal parameters of the hydrodynamic diameter (HD), polydispersity index (PDI), and zeta potential (ZP) were assessed after freezing.

Once the optimal freezing protocol was determined, Blank SLN formulations containing various concentrations of isolated glycerol (2.5, 5, 7.5, 10, and 20%) were prepared without NaDES to assess the specific contribution of glycerol to the cryoprotective effect. Samples were subjected to freezing at −20 °C (the freezing conditions selected were based on experimental findings). For this step, the formulation was prepared using the same methodology as previously described, and in the aqueous phase, the volume removed from water was proportional to the volume of glycerol added.

### Toxicity Assays with Zebrafish Embryos

For toxicity assays, zebrafish (*Danio rerio*) embryos were used, provided by the system of the Toxicological Genetics Laboratory (G-Tox) at the University of Brasília (UnB), where adult fish were kept in an automated recirculating water system supplied with activated charcoal-filtered and aerated water to eliminate chlorine (ZebTec, Tecniplast, Italy).

The embryos were selected and handled according to the OECD protocol for toxicity assessment: fish embryo toxicity (FET) test (OECD no. 236, 2013). After collection from the aquariums, the embryos were washed and screened based on morphological criteria following Kimmel et al.^28^ High-quality embryos were then transferred to 96-well microplates, with each well containing 200 μL of the respective test concentration. The tests were conditioned in a climatic chamber with conditions identical with those of the cultivation room. Test solutions were prepared by using the zebrafish system water.

All tests were performed in triplicate with a total of 60 organisms per concentration for 96 h. The concentrations tested were 0 (negative control, with system water), 0.003, 0.006, 0.013, 0.025, 0.05, 0.1, 0.2, and 0.4 mg/mL, considering the lipid component of SLN-Blank-NaDES, over a total exposure time of 96 h.

### Physicochemical Characterization of SLNs

#### Absorption and Fluorescence Spectroscopy

The absorbance and fluorescence spectra were plotted in the visible region of the electromagnetic spectrum. For the assay, transparent 96-well plates (absorbance) and black plates (fluorescence) were used, samples were diluted in a ratio of 1:4 (final concentrations of 5 μM), and 200 μL of each sample was deposited in triplicate.

The absorbance values were measured by spectral scanning between wavelengths of 350–750 nm with a resolution of 1 nm. Fluorescence was measured under excitation at 350 nm and emission between 360 and 750 nm, also with a resolution of 1 nm. The analyses were conducted using a Varioskan Spectrophotometer (Thermo Fisher, USA).

#### HD, PDI, and ZP

The analyses of HD and PDI were performed using dynamic light scattering (DLS), and ZP was assessed through electrophoretic mobility in a ZetaSizer SZ90 (Malvern, USA). For analysis, all samples were diluted to a 1:10 ratio in distilled water.

For the colloidal stability assays over time, the SLNs formulations were evaluated over a 365-day period under four storage conditions: room temperature (25 °C), refrigeration (4 °C), heating (37 °C), and freezing (−20 °C), with HD, PDI, and ZP parameters measured as mentioned above.

For the SLNs subjected to freezing, the samples were kept frozen for 24 h, regardless of the methodology employed. After this period, the samples were thawed at room temperature (25 °C) until complete melting of the material and subsequently diluted in distilled water at a 1:10 ratio for analysis using a Zetasizer SZ90 (Malvern, USA), as previously described.

### Methodologies for Concentrating SLNs

SLNs were subjected to drying procedures to concentrate nanoparticles in the sample through three different methodologies: vacuum drying and centrifugation, rotaevaporation, and lyophilization.

#### Vacuum Drying and Centrifugation

Triplicates of SLN-Blank, SLN-AlClPc, and SLN-Blank-NaDES samples were subjected to drying by vacuum and centrifugation in a Speed Vac Concentrator Savant SPD2010, and samples were taken every hour over a period of 7 h. After drying, the samples were diluted in distilled water (1:10) and analyzed using a ZetaSizer SZ90 instrument (Malvern, USA) to assess HD, PDI, and ZP.

#### Rotaevaporation

Triplicate samples of SLN-Blank, SLN-AlClPc, SLN-Blank-NaDES, and SLN-AlClPc-NaDES were subjected to solvent evaporation drying in a Rotaevaporator R-II at 250 mbar pressure for 5 h. After rotary evaporation, the samples were resuspended to their original volume and diluted in distilled water (1:10) for the assessment of HD, PDI, and ZP.

#### Lyophilization

Triplicate samples of SLN-Blank, SLN-AlClPc, SLN-Blank-NaDES, and SLN-AlClPc-NaDES were previously frozen at −20 °C and subjected to dehydration under vacuum and low temperatures in an L101 Lyophilizer for 72 h, until complete dehydration. Subsequently, the lyophilized samples were stored in a desiccator containing silica. For the assessment of HD, PDI, and ZP, the lyophilized samples were resuspended in distilled water to a final volume corresponding to that prior to lyophilization (1000 μL) and subsequently diluted at a 1:10 ratio for analysis using a Zetasizer SZ90 (Malvern, USA).

### Morphological Characterization

#### Transmission Electron Microscopy (TEM)

SLNs were initially diluted at a ratio of 1:200 (v/v) in distilled water, and 5 μL of the sample were deposited onto 400 mesh copper grids coated with Formvar film. After 24 h of deposition, the samples were contrasted with 2% osmium tetroxide vapor for 2 min and subsequently analyzed using a JEOL 1011 transmission electron microscope (Tokyo, Japan) operated at 80 kV.

#### Scanning Electron Microscopy (SEM)

Once the morphological characterization of SLNs had been performed by transmission electron microscopy (TEM), and given the study’s primary objective of evaluating the impact of NaDES incorporation and lyophilization on SLN morphology, scanning electron microscopy (SEM) analysis was focused on selected samples (with and without NaDES) and conducted exclusively on lyophilized and resuspended formulations, as these conditions represent the most relevant structural transitions explored in this work.

Thus, 400 mesh copper grids coated with the Formvar film were placed on carbon tape in metallic stubs, and then, the samples were deposited. The lyophilized SLNs were previously resuspended and diluted at a ratio of 1:200 (v/v) in distilled water, deposited in a volume of 5 μL onto the grid on the stub and were subsequently contrasted with 2% osmium tetroxide vapor for 5 min. After 24 h of deposition, the samples were coated with gold for 1 min in an SCD 500 Metalizer (LEICA, Germany) and then analyzed using a JSM-7001F Scanning Electron Microscope (JEOL, Japan).

### Fourier-Transform Infrared Spectroscopy (FTIR)

Samples were prepared and deposited on a diamond crystal in distinct physical states due to their specificities, which are (i) solid (citric acid, choline chloride, and AlClPc), (ii) liquid (glycerol, NaDES, physical mixtures, and nonlyophilized nanocarriers), and (iii) paste-like (lyophilized nanocarriers). The spectra were acquired in attenuated total reflectance (ATR) mode using the Vertex 70 spectrophotometer (Bruker Corporation, USA) and processed with the OPUS 7.2 software (Bruker Corporation, USA) in the range from 4000 to 500 cm^–1^, with a resolution of 4 cm^–1^ and 32 scans. The data were analyzed using OriginPro 2015 software (OriginLab Corporation, USA).

### Statistical Analysis

The statistical packages GraphPad Prism 10.0 and Sigma Plot 14.0 were used for statistical analysis. Two-way ANOVA was used to detect differences between groups for normally distributed data sets. In cases of nonnormal distributions, the Kolmogorov–Smirnov test for normality and Levene’s test for variance homogeneity were applied, followed by the Kruskal–Wallis test. Dunnett or Dunn’s test (for parametric or nonparametric tests, respectively) were used to detect significant differences between the tested concentrations and the control (*p* < 0.05).

## Results and Discussion

### Formulation of the NaDES

The use of terms related to eutectic systems, such as deep eutectic solvents (DES) and natural deep eutectic solvents (NaDES), varies in the literature, depending on their characteristics, which are often influenced by the degree of dilution of these systems in aqueous media. Although classically defined by a very low eutectic melting point, recent evidence shows that even when diluted in water, NaDESs retain relevant functional properties, due to the hydrogen bonding network between donors and acceptors, which forms a pseudoionic fluid arrangement capable of retaining water and stabilizing solutes.^29^

Joules et al.^30^ demonstrated that diluted saccharide-based NaDESs mixtures exhibit reduced water activity and stronger hydrogen bonding compared to similar noneutectic systems, suggesting the persistence of cooperative interactions even in aqueous media. Also, they demonstrated that even extreme dilutions, such as 90% in water, although they alter the physicochemical properties, do not entirely lose their identity as NaDESs. Similarly, reports on biological processes indicate that water in NaDESs is “strongly retained within the liquid and does not evaporate easily.”^20^

There are clear precedents for aqueous NaDESs being successfully used in cell cryopreservation without the strict need to maintain the solid eutectic phase. Castro et al.^31^ reported the first application of trehalose/glycerol NaDESs as cryoprotectants by water crystallization reduction, decreasing ice-induced damage and achieving performance equal to or superior to DMSO. More recently, Jesus et al.^21^ and Hornberger et al.^32^ developed formulations in which NaDESs were diluted in buffered aqueous media at typical cryoprotectant concentrations, resulting in solutions initially containing NaDES at 10–20% in an aqueous medium, and subsequently further diluted with 50% water (w/v), yet still retaining the key physicochemical properties characteristic of eutectic systems. In particular, Hornberger et al.^32^ demonstrated that 10% of a trehalose: glycerol NaDES in Normosol-R prevented excessive ice formation and enabled cryopreservation of cells down to −20 °C, indicating that the hydrogen bond network of the NaDES activates cryoprotective functions even at high dilution. These studies demonstrated post-thaw cell recovery comparable to or better than standard mixtures. Therefore, even though the “ideal” eutectic structure may be altered by the addition of water, the functional hydrogen-bonding network is preserved, ensuring the system’s functionality.

For these reasons, many authors refer to such highly diluted mixtures as “DES-like” or “pseudo-DES” systems. For example, Ghigo et al.^33^ describe similar reaction conditions as occurring in “DES-like systems.” These perspectives support the treatment of media such as NaDES in the literature, highlighting their emergent properties, such as dual solvation behavior and the stabilizing effects typically associated with deep eutectic systems.

In other words, these systems are not conventional aqueous solvents but solutions in which the nature of NaDES (components and hydrogen bonding arrangement) predominates in governing its behavior. From a chemical perspective, NaDESs act as pseudoionic fluids: they exhibit dual affinity, capable of solubilizing both hydrophilic and hydrophobic compounds. Therefore, the term “functional eutectic solvents” or “functional eutectic systems” has also been proposed to emphasize that their roles (as plasticizers, cryoprotectants, stabilizers) persist even when the typical crystalline eutectic structure no longer exists.^20^

Research groups highlight that the functional nature of NaDESs transcends the necessity of a single eutectic point. Abbott et al.^34^ pioneered the definition of mixtures containing water and salts in liquid form as “pseudo-binary DES-like.” Thus, a partially altered structure is tolerated as long as the system continues to fulfill its role: serving as a specialized solvation medium.

This “cumulative reinforcement” explains why diluted NaDES exhibit water with reduced activity while still maintaining a high latent heat of fusion.^30^ In summary, there is experimental evidence to assert that despite the absence of a solid “eutectic salt,” the cryoprotective action arises from the natural solutes themselves (osmolytes) acting in synergy, which aligns with one of the central concepts of NaDES.

Based on the theoretical framework presented, recent literature may support the use of the NaDESs concept in aqueous SLN solutions without the requirement to demonstrate a “solid eutectic point.” This has allowed various studies to adopt terms such as “NaDES-based” provided that the system’s behavior is justified through functional analogy.^35–39^

Thus, in the context of the present research, the term “NaDES” refers to the diluted NaDES used on SLN formulations, which underwent initial dilution with 25% water, followed by a further dilution during the SLN formulation step, at which point the solvent became highly diluted as an inherent result of the nanoparticle preparation process. Under these conditions, the SLN formulations contained 12.5% of NaDES, a concentration similar to that reported in previous studies, such as those by Joules et al.^30^ and Hornberger et al.,^32^ as previously mentioned. Therefore, this terminology more accurately reflects a system based on a natural deep eutectic solvent mixture and better specifies the characteristics of the solvent employed in our study.

Initially, to confirm the proper formation of the NaDES, Fourier-transform infrared spectroscopy (FTIR) was employed to analyze the chemical functional groups and identify intermolecular interactions among the system’s components. The spectral profile of NaDES was analyzed, along with the isolated constituents involved in its formulation: glycerol, citric acid, and choline chloride (Supporting Information: Figure S1).

A complex composition is observed, and the dashed lines (1– 12) represent the peaks shared between NaDES and its constituents. The peaks found are consistent with the literature.^22^ The broad band at 3321 cm^–1^ (1) is associated with the ν­(OH) group, while the narrow peaks at 2934 (2) and 2890 cm^–1^ (3) correspond to the ν_as_(CH_2_) and _νs_(CH_2_) groups. The signatures at 1721 and 1646 cm^–1^ (5) may be related to the ν­(C=O) stretching vibration. In the fingerprint region (1500–500 cm^–1^), the strong and narrow peak at 1035 cm^–1^ (10) may correspond to the ν_as_(C–C–O) stretching vibration, indicative of both glycerol and choline chloride. The additional medium and weak peaks at 1411 (6), 1331 (7), 1205 (8), 1107 (9), 991 (11), and 860 cm^–1^ (12) can be attributed, in this order, to δ­(C–O–H), ρ­(C–O–H), ν­(C–C–O), δ­(OH), δ­(OH), and ν_s_(C–C–O).

The formation of NaDES can be indicated by the distinct spectral profiles of the solvent compared to its individual components, with a predominant influence from glycerol, as reported by Elderderi et al.^40^ According to Ghaedi et al.^41^ and Ozturk et al.,^42^ high-intensity peaks in the 3300 cm^–1^ band (1) suggest the formation of hydrogen bonds between the electron donor and acceptor components of NaDES, namely glycerol, citric acid, and choline chloride. These findings corroborate with those of Silva et al.,^22^ who investigated the same NaDES formulation.

The ternary combination of choline chloride, citric acid, and glycerol exhibits high biocompatibility and is widely employed in the pharmaceutical industry, both as an excipient and as an extraction medium.^43^ This NaDES-based system formulation has demonstrated proven efficiency in the extraction and stabilization of anthocyanins,^22^ which are phenolic compounds possessing conjugated aromatic structures similar to the phthalocyanines used in the present formulation.

Although the literature on the application of NaDESs in the solubilization or stabilization of phthalocyanines remains limited, this structural resemblance suggests a potential chemical compatibility between the selected NaDES and AlClPc, which may enhance its dispersion and stability.^44^

In the case of SLNs containing photosensitizers, the NaDES-based system composed of choline chloride:glycerol:citric acid (0.5:2:0.5) was incorporated as the internal solubilizing medium not only due to its extractive efficiency already demonstrated in the literature but primarily because of a set of intrinsic physicochemical properties of its components and their specific molar ratio, which confer functional robustness to the system. In this formulation, glycerol (a donor of three hydroxyl groups) and citric acid (bearing multiple carboxylic groups) strongly interact with choline chloride, resulting in a eutectic system with a significantly depressed melting point, near-zero volatility, and tunable rheological behavior.^45^ Such structural characteristics may confer superior colloidal stability, particularly in processes involving freeze–thaw cycles, given that glycerol—one of the NaDES components—is a well-known classical cryoprotectant due to its ability to penetrate colloidal systems, inhibit ice crystal formation, and stabilize dispersed systems during freeze–thaw cycles.^32,46^

The molar ratio employed, with glycerol and citric acid acting as hydrogen bond donors and choline chloride as the acceptor, maximizes intermolecular interactions and promotes a dynamic hydrogen-bonded network within the continuous phase. This structural organization restricts water molecule mobility during the cooling process, thereby inhibiting ice crystal nucleation, a mechanism that underlies the intrinsic cryoprotective effect of the system and contributes to enhanced nanoparticle stability during freeze–thaw cycles.^47^

Recent studies, such as Rasool et al.,^25^ have also demonstrated that compounds based on this type of NaDES are capable of effectively modulating rheological properties, increasing the yield point-to-plastic viscosity ratio in model fluids—which, in our case, translates directly into improved control over photosensitizer dispersion and enhanced colloidal stability of the formulation. In practical terms, the 0.5:2:0.5 molar ratio represents a balance point between fluidity and structural integrity, enabling efficient formulation processing without compromising functional performance or toxicological safety.

Moreover, the composition of the NaDES-based system employed in this study was selected based on criteria of industrial scalability and biological safety, as all components are substances classified as Generally Recognized As Safe (GRAS), cost-effective, and widely available, factors that greatly facilitate the transition to large-scale production. The simplicity of its preparation, the absence of critical purification steps, and its compatibility with lipophilic active compounds further underscore its technological value.^48^

### Incorporation of NaDES into the Formulation of SLNs

Although previous studies have described the use of NaDESs in nanoparticle synthesis, particularly for metallic nanoparticles,^49–51^ as well as in the solubilization of extracts during the formulation process of nanostructured lipid carriers,^52^ no reports have been found in the literature detailing SLNs formulation methodologies using these solvents for cryoprotection. Thus, this research represents an innovation in the application of NaDESs and the study of SLNs, paving the way for the use of sustainable alternatives in industrial scaling processes, integrating nanotechnology, and green chemistry.

For this study, formulations of SLNs associated with an NaDES were developed based on sustainable and environmentally friendly processes. The aim is to explore the cryoprotective properties of these solvents when applied to SLNs.

In this context, the development of SLNs builds upon formulations previously established by Mello et al.,^27^ introducing the innovative incorporation of NaDES to improve industrial efficiency. The research describes the development of two novel SLN systems: one without active compound loading (SLN-Blank-NaDES) and the other loaded with the drug AlClPc (SLN-AlClPc-NaDES).

Initially, to understand the effects of the solvent addition in nanoparticle formulation, NaDES was added at different concentrations to the SLN-Blank formulation. Only after selecting the formulation with the most suitable colloidal parameters the solvent was added to the SLN containing the photosensitizer (SLN-AlClPc). Figure 1 illustrates the colloidal parameters of HD and PDI for the SLN-Blank formulations containing different concentrations of NaDES.

**Figure 1:**
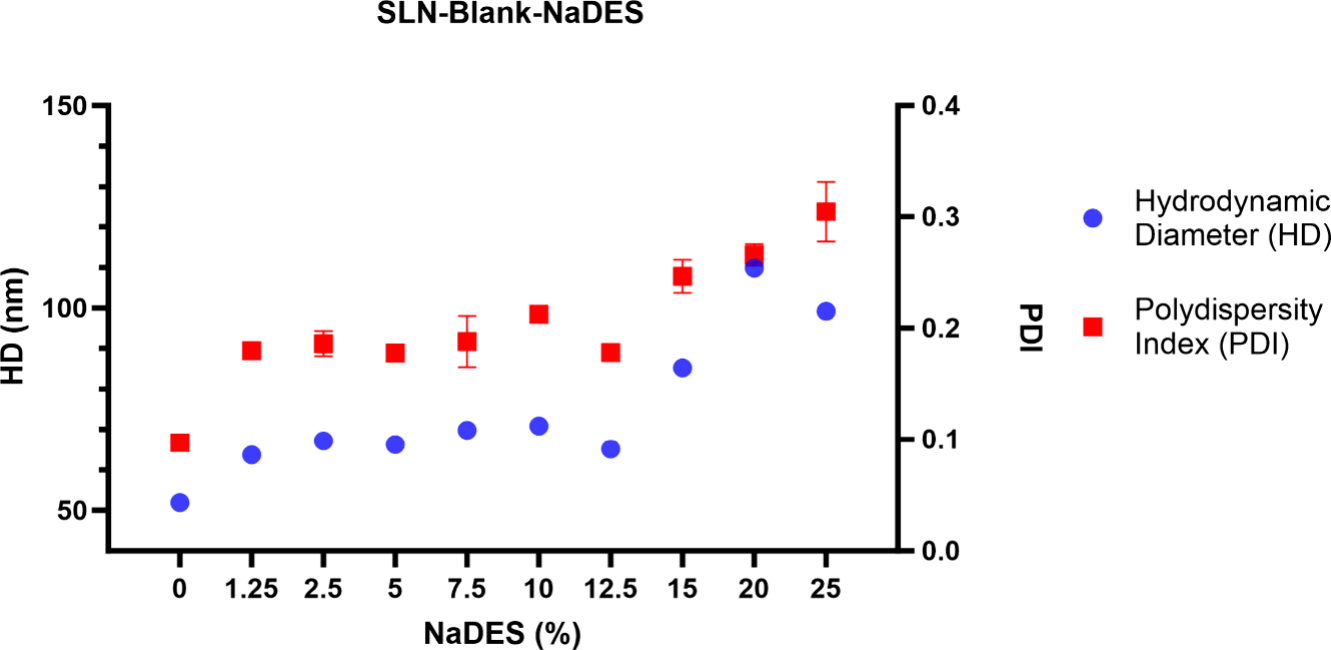
Colloidal parameters of SLN-Blank and SLN-Blank-NaDES formulations with different NaDES concentrations. At 0%, the formulation is a nanoparticle with no solvent addition, representing SLN-Blank. The graph represents the colloidal parameters of the HD and PDI of SLN-Blank and SLN-Blank-NaDES formulations. Formulations containing 0, 1.25, 2.5, 5, 7.5, 10, 12.5, 15, 20, and 25% solvent were prepared. For each amount of NaDES added, an equivalent volume of water was removed. The data represent the mean ± SEM of triplicates of the formulations.

The solvent at low concentrations (1.25–12.5%) leads to an increase in the HD of the particles, rising from 50 nm to approximately 70 nm, which corresponds to 40% of the original size. A similar trend is noted for the PDI, reaching approximately 0.180. The PDI reflects the size homogeneity of the particles in a sample. For lipid nanoparticles, PDI values ≤0.3 are considered acceptable, indicating populations with homogeneous sizes.^53,54^ In the formulated SLNs, these colloidal parameters remain stable up to an NaDES concentration of 12.5% (v/v), demonstrating no significant changes in the formulation.

However, at higher solvent concentrations (15–25%), there was a significant increase in the HD of the particles, representing more than twice their original size. A similar effect was observed for the PDI, which reaches very high values (>0.3), indicating that the use of NaDES in SLNs formulations at elevated concentrations can interfere with the polydispersity of the particles. Therefore, the SLN-Blank-NaDES formulation containing 12.5% NaDES was the one that supported the highest solvent incorporation without inducing significant changes in HD and PDI. Once the maximum incorporated amount of NaDES supported by the formulation was determined, we aimed to identify the optimal concentration within this range to enhance the cryoprotective effect on the nanoparticles

### Evaluation of the Cryoprotective Potential of NaDES in SLNs Formulation

Due to the stresses that occur during the freezing process, coupled with ice crystal formation, SLNs tend to form aggregates, leading to an increase in particle size and the rupture of their structure. This results in degradation and renders certain protocols unfeasible, limiting the applications of such nanoparticles. Therefore, the use of cryoprotectants in SLNs formulations is necessary to prevent these issues.^11,55^

It has been demonstrated that primary metabolites, such as sugars, organic acids, and choline derivatives, play a crucial role in protecting against extreme cold conditions in plants and animals. In biological systems, these components can interact with themselves and water, leading to lower crystallization temperature, thereby reducing or preventing the formation of ice crystals. These crystals, when formed, can disrupt biological structures and degrade the sample. Thus, NaDES, being a mixture of these naturally occurring compounds, exhibits similar properties and acts as a vitrifying agent, forming a protective barrier that promotes sample vitrification and preserves its integrity after freezing.^31,56,57^

Considering the cryoprotective properties historically exhibited by NaDES and the need to freeze SLNs for certain protocols, such as lyophilization and improved storage, this study investigated the cryoprotective potential of this solvent in different types of freezing for SLN-Blank-NaDES formulations.

It is important to mention that the freezing tests were conducted with formulations containing up to 12.5% NaDES, once SLNs with higher concentrations exhibited unsatisfactory colloidal parameters, even before freezing. The results are presented in Figure 2.

**Figure 2:**
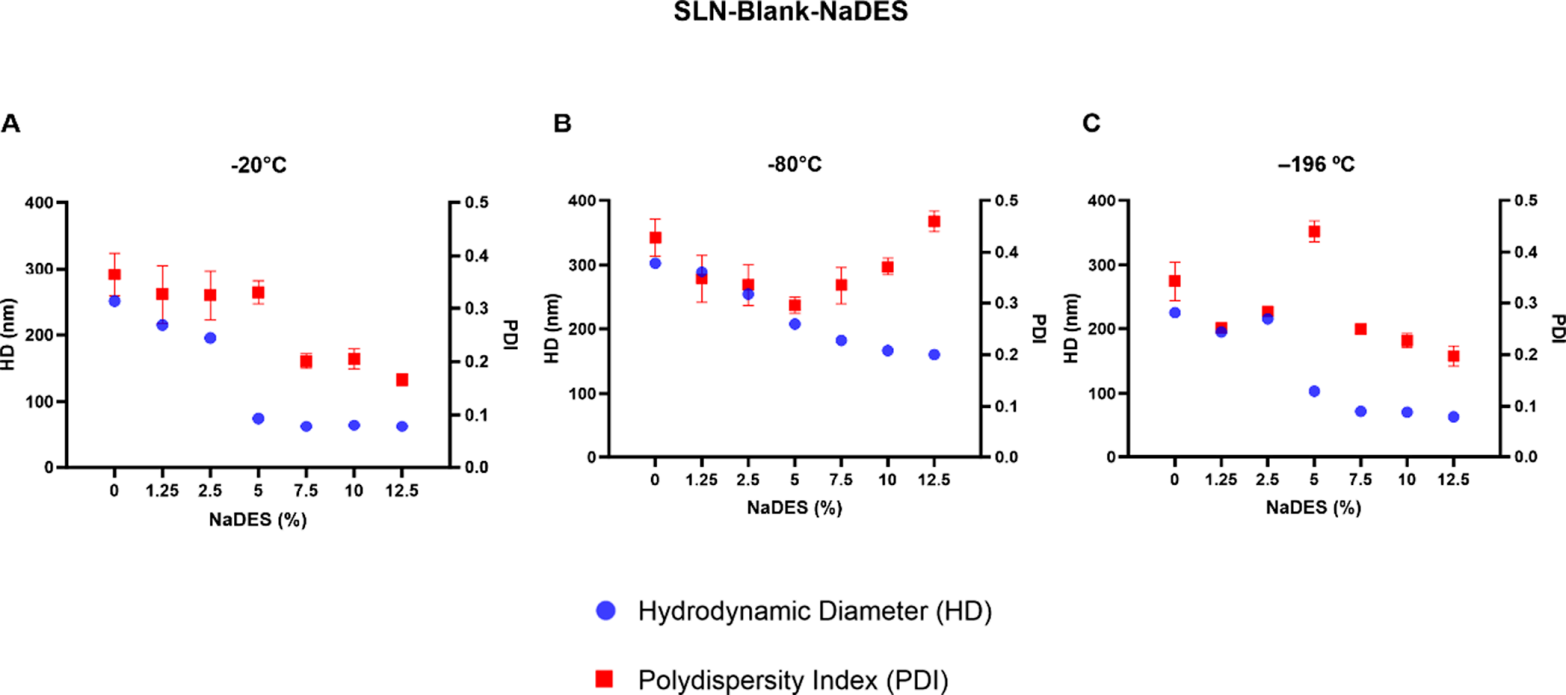
Colloidal parameters of NLS-Blank and NLS-Blank-NaDES formulations with different NaDES concentrations after they were subjected to different freezing methods. The graph represents the colloidal parameters of HD and PDI of SLN-Blank and SLN-Blank-NaDES formulations subjected to three types of freezing: slow freezing at −20 °C (A), rapid freezing at −80 °C (B), and ultrafast freezing with liquid nitrogen at −196 °C (C). At 0%, the formulation is a nanoparticle with no solvent added (SLN-Blank). The data represent the mean ± SEM of triplicates of the formulations.

The SLN-Blank, which contained no NaDES in its formulation (0%), was destabilized in all freezing methods analyzed (Figure 2A–C), with its HD increasing to 250–300 nm, approximately six times larger than the initial size of 50 nm (Figure 1). The PDI of these particles was also elevated, reaching 0.4 after freezing.

For rapid freezing at −80 °C (Figure 2B), none of the formulations resisted degradation, with significant increases in both HD and PDI (PDI ≥ 0.3). In contrast, freezing at −20 and −196 °C showed a very similar profile (Figure 2A,C), where SLNs formulations with higher concentrations of NaDES (7.5–12.5%) performed better during freezing, as their colloidal parameters remained virtually unchanged. This was not the case for formulations with lower NaDES concentrations (1.25–5%), which were easily degraded during freezing, exhibiting significant changes in both HD and PDI parameters.

The formulation of SLN-Blank-NaDES containing 12.5% remained stable under both slow freezing (−20 °C) and ultrafast freezing (−196 °C). However, due to its lower cost and greater feasibility, the slow freezing method at −20 °C was selected to proceed with the subsequent protocols involving the freezing of the SLNs. Therefore, based on the colloidal profile presented, the formulation of SLN-Blank-NaDES containing 12.5% of the solvent, as it showed the highest solvent incorporation while maintaining colloidal parameters, was chosen to proceed with the assays, along with the slow freezing method at −20 °C.

The variation in response to different freezing methods can be primarily attributed to the speed at which they occur, which also influences the rate of crystal formation.^58^ However, this should not be the only factor since rapid freezing at −80 °C resulted in particle degradation, whereas freezing at −196 °C, despite being instantaneous, maintained the properties of the SLNs at higher solvent concentrations. This suggests that the cryoprotective capacity in the formulation was defined by the solvent amounts, as higher quantities lead to a greater stability of the SLNs during the process. Similar results were obtained by Lee et al.^59^ when evaluating the impacts of freezing rate on the resuspension of nanocrystals after lyophilization.

To investigate whether the observed cryoprotective effect was attributable to the NaDES as a whole or specifically to the presence of glycerol, given that glycerol itself exhibits well-known cryoprotective properties,^57^ blank SLN formulations containing different concentrations of isolated glycerol (SLN-Glycerol) were prepared in the absence of NaDES and subjected to freezing at – 20 °C, condition previously justified (Supporting Information: Figure S2).

Considering the molar ratios used in the preparation of NaDES diluted in water, the glycerol content in the system corresponds to 39.5%. Based on the amount of NaDES employed in the SLN formulations, the estimated amount of glycerol incorporated was approximately 1.97 g (∼2 mL). Analysis of the SLN-Blank formulations containing isolated glycerol revealed that, after freezing, the samples exhibited degradation, as evidenced by an increased HD and PDI at glycerol concentrations of 2.5, 5, and 7.5%. These results suggest that glycerol exerts a significant cryoprotective effect only at concentrations of 10% or higher, corresponding to approximately 4 g, more than twice the amount present in the NaDES-based formulations.

These results demonstrate the potential of the NaDES used in this study to act as a cryoprotectant in SLNs formulations, protecting the particles from degradation during freezing, which supports the findings of cryoprotective activities of these solvents^56^ while also broadening their applications in nanoparticle cryoprotection.

### Ecotoxicological Evaluation of Zebrafish Embryo Exposure to SLNs Containing NaDES

After the cryoprotective potential of the solvent in the developed formulations was confirmed, assays were conducted to evaluate the biological toxicity of the nanoparticle developed in this study on zebrafish embryos, aiming to understand the possible environmental impacts of the formulation before proceeding with further assays.

The industrial-scale application of nanoparticles has experienced significant growth in recent years. This expansion raises concerns about the potential environmental risks associated with the use of nanomaterials across various industrial activities. Therefore, assessing the ecotoxicological impacts of nanomaterial exposure is essential to ensure their safe and sustainable application.^60^ The zebrafish is considered a reference model in environmental toxicology research, primarily due to its small size, high reproductive rate, and rapid embryonic development. These characteristics make it an ideal species for rapid screening assays to assess the toxicity of nanoparticles.^61^ An overview of the embryotoxicity assay is shown in Figure 3.

**Figure 3:**
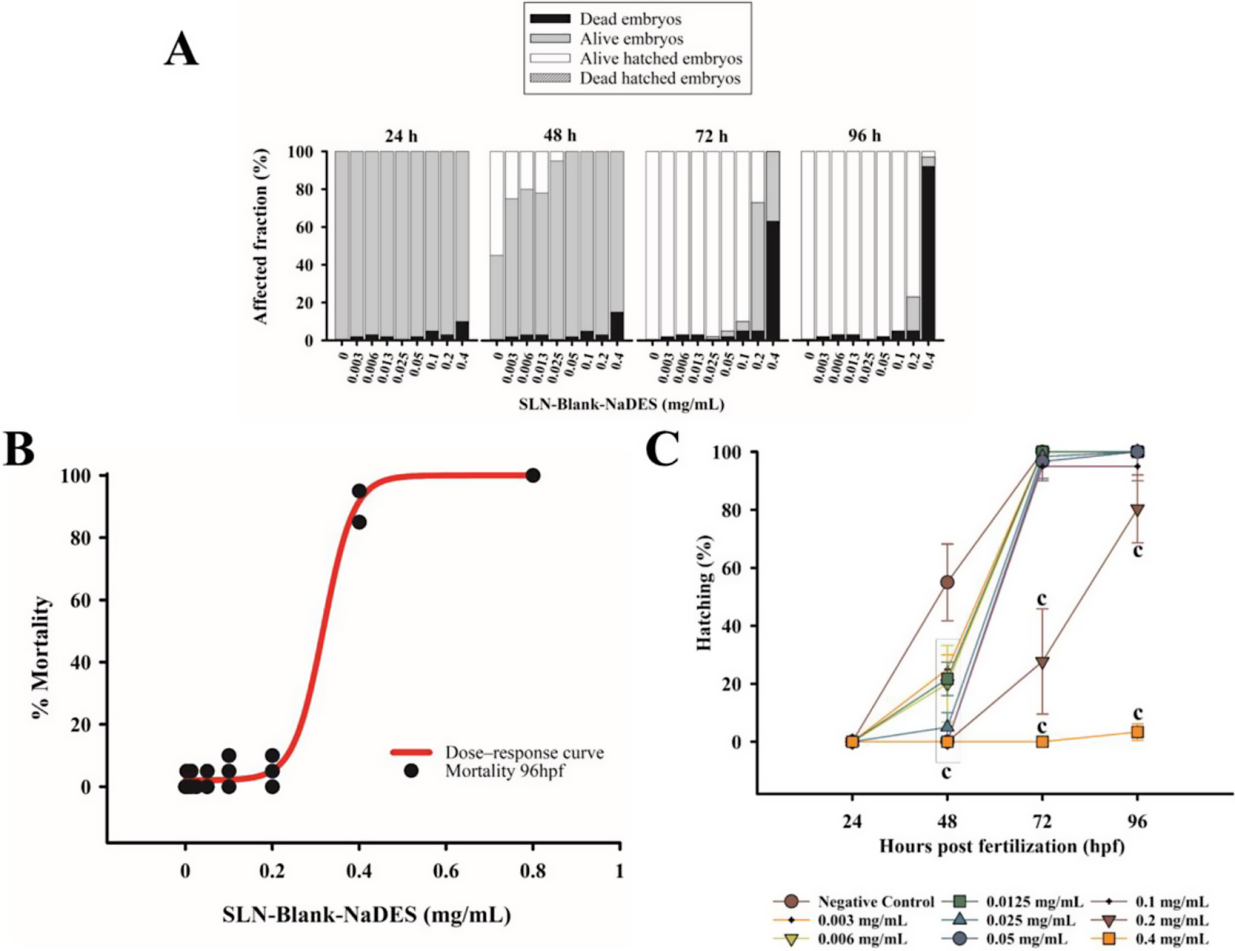
Graph representing the affected fraction (%) of embryos exposed to different concentrations of SLN-Blank-NaDES (milligrams per milliliter) over time. The bars indicate the proportion of dead embryos (black), alive embryos (gray), alive hatched embryos (white), and dead hatched embryos (hatched) (A). Dose–response curve (mortality) of organisms exposed for 96 h to SLN-Blank-NaDES at concentrations of 0 (negative control); 0.003; 0.006; 0.013; 0.025; 0.05; 0.1; 0.2; 0.4; 0.8 mg/mL, with an LC_50_ of 0.3180 ± 0.0122 - Model: Sigmoid – four parameters. *R* ^2^ 0.99 (B). Hatching percentage (%) of embryos exposed to different concentrations of SLN-Blank-NaDES (mg/mL) over hours postfertilization, hpf). The letter ″c″ indicates statistically significant differences compared to the negative control (*p* < 0.001). Error bars represent the standard deviation (C). NaDES concentration in the initial test sample was 0.0005%.

The lethal concentration for 50% of the studied population (LC50) for SLN-Blank-NaDES was determined (Figure 3B), with a value of 0.3180 mg/mL (four-parameter logistic, *R* ^2^ = 0.99), indicating relatively moderate toxicity compared to previous studies using similar nanoparticles.^48,49^

During the 96 h test, no lethal (mortality) or sublethal effects (behavioral changes, hatching, heart rate, and embryonic development) were observed in the negative control, which exhibited normal development as described by Kimmel et al.^28^ After 24 h of exposure, mortality was observed at the highest concentrations, progressively increasing throughout the 96-h period. Within the first 48 hpf, a significant delay in hatching was observed across all tested concentrations, persisting throughout the test at the two highest concentrations. However, despite these hatching delays, embryos at lower concentrations successfully hatched alive (Figure 3C). Furthermore, although hatching was delayed, the posthatching mortality rate remained zero up to a concentration of 0.4 mg/mL.

In contrast, a similar study conducted by Schuh et al.,^62^ using a different NaDES in nanostructured lipid carriers, reported the hatching of dead embryos at 72 h and at nanoparticle concentrations lower than those in the present study, as well as higher mortality rates at earlier time points.

In addition to evaluating the hatching rate, it is also possible to identify significant sublethal effects (Figure 4) over the 96 h test period, such as yolk sac and pericardial edema, blood stasis, spinal curvature abnormalities, malabsorption syndrome, and yolk sac darkening. These effects were more pronounced at higher concentrations (0.2 and 0.4 mg/mL), with an increasing incidence over time,^63^ suggesting that exposure to SLN-Blank-NaDES may impair embryonic development, especially at higher concentrations (Figure 4A).

**Figure 4:**
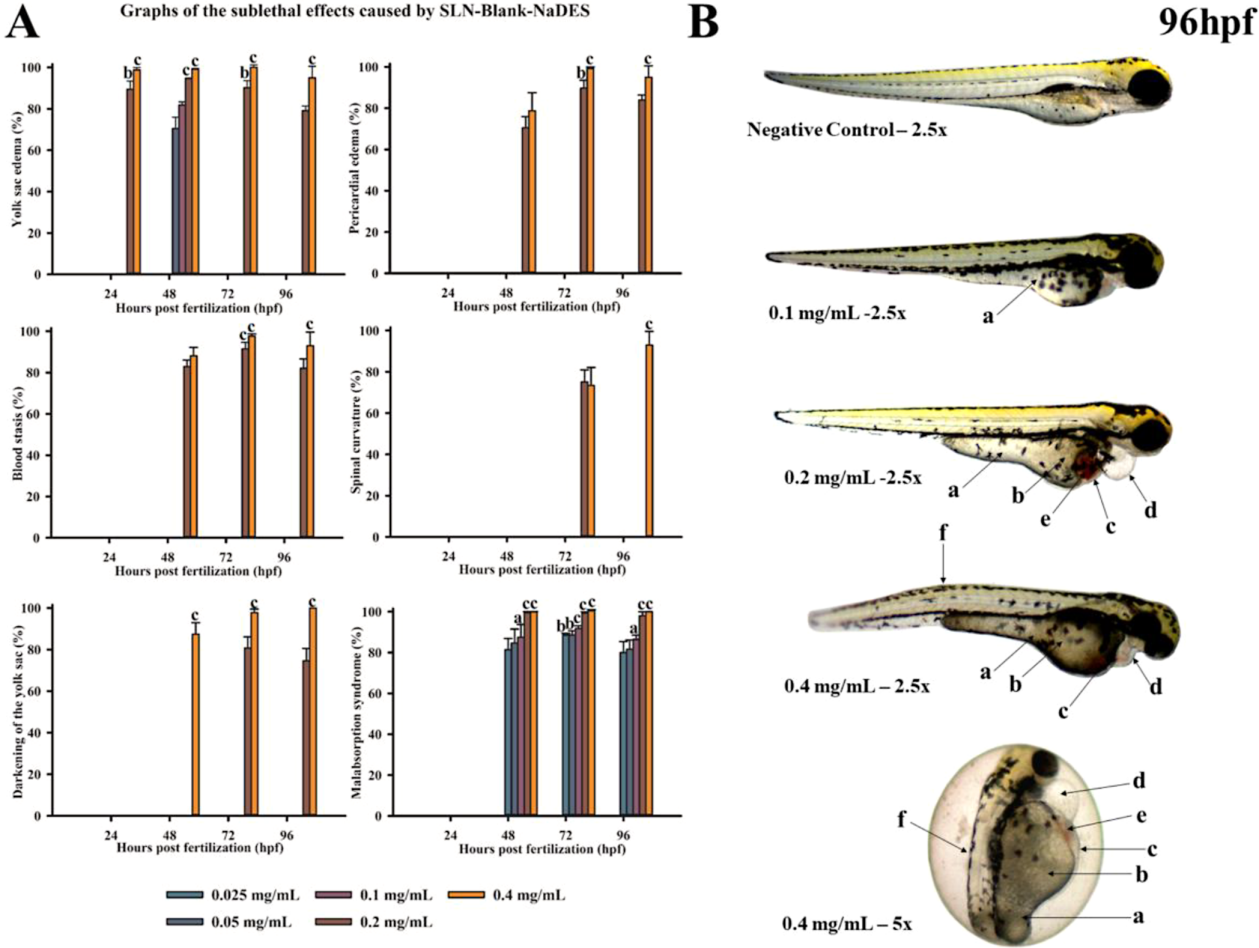
Graphs illustrating the sublethal effects of exposure to different concentrations of SLN-Blank-NaDES (0.025, 0.05, 0.1, 0.2, and 0.4 mg/mL) over 96 h postfertilization (hpf). Error bars represent the standard deviation. Statistically significant differences compared to the negative control are indicated by the letters: ″a″ (*p* < 0.05), ″b″ (*p* < 0.005), and ″c″ (*p* < 0.001) (A). Photo documentation of zebrafish embryos after 96 h of exposure to SLN-Blank-NaDES, illustrating organisms exposed to SLN-White-NaDES at concentrations of 0 mg/mL (negative control), 0.1, 0.2, and 0.4 mg/mL (embryos and larvae). (a) Malabsorption syndrome; (b) yolk sac darkening; (c) yolk sac edema; (d) pericardial edema; (e) blood stasis; and (f) spinal curvature abnormalities, a sublethal effect with statistically significant differences. The images were captured at 2.5× and 5× magnification (B). NaDES concentration in the initial test sample was 0.0005%.

Furthermore, in Figure 4B, the main sublethal effects can be observed after 96 h of exposure, such as yolk sac and pericardial edema, blood stasis, spinal curvature abnormalities, malabsorption syndrome, and yolk sac darkening. These effects may be attributed to the interaction of SLN-Blank-NaDES with essential physiological processes critical for embryonic development, such as blood circulation and nutrient transport.^64,65^ High concentrations (0.2 and 0.4 mg/mL) could induce oxidative stress,^66^ causing cellular damage and compromising the integrity of cell and vascular membranes. Additionally, the accumulation of toxic substances might affect the developing nervous and muscular systems,^67,68^ leading to the observed abnormalities. The increasing incidence of these effects over time suggests that prolonged exposure to SLN-Blank-NaDES negatively impacts embryonic development, especially at higher concentrations.

The results obtained from the embryotoxicity assay demonstrate a dose-dependent toxicity profile and highlight the critical role of concentration and exposure time on the mortality rate of embryos exposed to SLN-Blank-NaDES.

Despite the relevance of the ecotoxicological assessment performed in this study, it is important to acknowledge that only the formulation containing NaDES was evaluated, which constitutes a limitation of the present study. Nevertheless, the formulation tested represents the most critical and representative condition for a preliminary environmental safety assessment, especially considering that NaDES is not a conventional component in SLN formulations. These findings provide a data foundation for future research in ecotoxicology related to the use of NaDESs in SLNs and emphasize the necessity for comprehensive evaluations to better elucidate the toxicity mechanisms involved.

Additionally, they emphasize the importance of establishing safe environmental usage conditions for the developed nanoparticles while encouraging the advancement of more sustainable and environmentally responsible materials.

### Effect of NaDES Incorporation of the Physicochemical Properties and Stability of the Formulation

After assessment of the feasibility of applying the developed nanoparticles as a scalable and environmentally low-impact tool, the physicochemical properties of the complete system were further investigated in detail.

In the first instance, to understand the effects of NaDES addition on the photophysical properties of the SLN formulations, absorption and fluorescence spectra were plotted in the visible region of the electromagnetic spectrum. Figure 5 illustrates the absorbance and fluorescence profiles of the analyzed samples.

**Figure 5:**
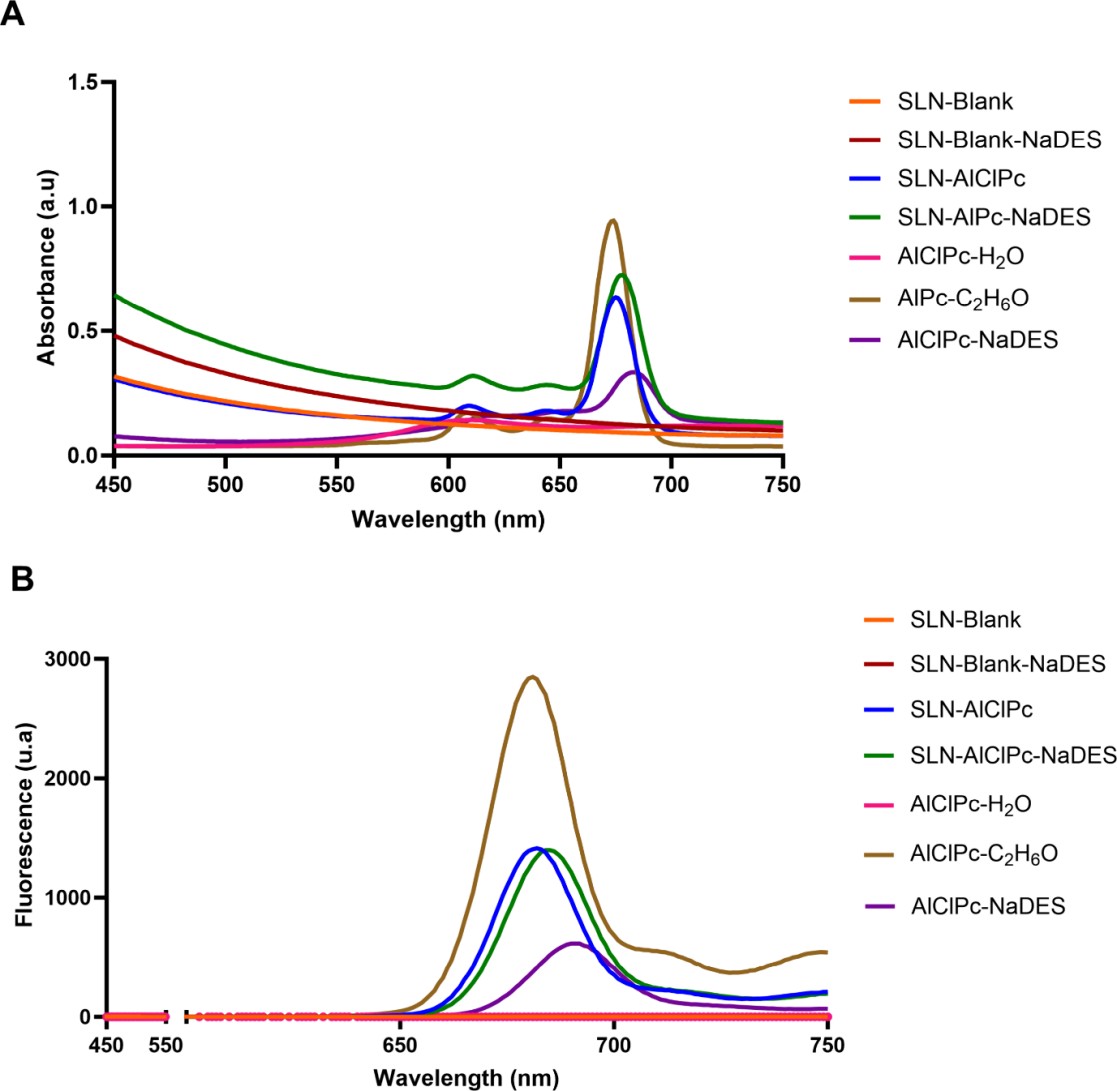
Absorbance spectrum (A) and fluorescence spectrum (excitation at 350 nm) (B) of SLNs without NaDES and SLNs with NaDES, suspended in aqueous solution, as well as AlClPc solubilized in control medium (Water, Ethanol, and NaDES). All samples are at a concentration of 5 μM, considering AlClPc as a reference.

As expected, the results do not show specific absorption peaks or fluorescence emission in the analyzed range for SLN-Blank and SLN-Blank-NaDES, with the spectra remaining at the baseline. However, it is observed that due to the turbidity of the samples, composed of nanoparticles, there is increased absorption in the initial range of the spectrum, which is likely attributed to the lipid compounds present, and this was not observed for the free AlClPc samples.

When comparing the spectra of SLN-AlClPc and SLN-AlClPc-NaDES, it is notable that both exhibit similar absorption spectra (674 and 678 nm, respectively) and absorption intensity, as well as similar fluorescence emission (682 and 654 nm, respectively). This similarity between the spectra suggests that the addition of the solvent to the nanoparticle formulation did not result in changes that would compromise the photophysical properties of the formulations. These results are consistent with findings in the literature,^69^ making this an advantageous aspect of incorporating NaDESs into SLNs.

For the spectra of free AlClPc, it is observed that AlClPc does not exhibit absorption or fluorescence emission peaks when dispersed in water. This occurs because the drug is highly hydrophobic and undergoes supramolecular aggregation in water. As a consequence, free AlClPc dispersed in aqueous media shows no detectable absorption or fluorescence, indicating that it remains aggregated and inactive.^70,71^ AlClPc solubilized in ethanol, on the other hand, showed more intense absorption and fluorescence emission peaks around 680 nm, a characteristic of this molecule, as it is in a medium in which solubilization occurs easily.

Studies report that the poor water solubility of AlClPc represents a significant limitation for clinical applications, as its aggregated state prevents the formation of monomeric excited species required for singlet oxygen generation, thereby reducing its photodynamic activity. Additionally, the difficulty in dissolving the drug compromises its biodistribution and hinders the achievement of therapeutically relevant doses.^27^ To overcome these limitations, various delivery systems have been investigated to maintain AlClPc in its monomeric form and improve its distribution.

To further investigate the behavior of AlClPc in the studied solvent, the AlClPc spectrum in NaDES was plotted at the same concentration as those of the other solutions. An absorption peak around 680 nm and a fluorescence emission peak near 690 nm were observed. Although the intensity is low, these peaks may suggest potential solubilization of AlClPc by NaDES. This is particularly noteworthy, as AlClPc is a highly hydrophobic molecule, typically solubilized only in toxic solvents.^72^ This finding represents a significant innovation in the present study, paving the way for further investigations into the specific NaDES used.

There are still a few published studies directly correlating NaDES with phthalocyanines. However, analogous evidence indicates that NaDES can significantly enhance the solubilization of hydrophobic compounds and photosensitizers. A notable example is the anticancer drug paclitaxel (completely insoluble in water), which achieved solubility several times higher in an NaDES composed of glucose and choline chloride (1:1 molar ratio) compared to pure water.^73^ Similarly, Wikene et al.^74^ identified two NaDES formulations with superior solubilizing capacity for the porphyrinic photosensitizer 5,10,15,20-tetrakis­(4-hydroxyphenyl)­porphyrin, which also exhibited greater photostability than in methanol. This effect is believed to be related to the semicrystalline-like structure of NaDES, which forms organized hydrogen-bonded networks that create a protective coating around the photosensitizer.

The presence of NaDES tends to condition the lipid interface, facilitating a more homogeneous distribution of AlClPc within the SLN matrix, rather than allowing its migration to the aqueous phase or aggregation. This molecular environment modification also reduces the characteristic aggregation behavior of AlClPc. In general, aromatic photosensitizers have a strong tendency to self-associate via π–π interactions, which impairs their dispersibility. We hypothesize that the NaDES components intercalate between AlClPc molecules, preventing stacking and keeping them separated.^52^ Analogous studies support this hypothesis: curcumin (another aromatic photosensitizer) when dissolved in an NaDES, formed uniform supersaturated solutions in aqueous media.^75^

These findings suggest that NaDES can effectively disperse photosensitizers structurally analogous to phthalocyanines. Accordingly, it is expected that a suitably chosen NaDES, such as choline chloride: glycerol: citric acid, may similarly enhance the solubility or dispersion of AlClPc, which may explain the results obtained with the drug solubilized exclusively in the NaDES.

Although it was observed that the addition of NaDES to the SLN formulation did not result in significant changes in the optical behavior of the sample, as evidenced by the absorbance and fluorescence results, colloidal stability was further investigated to determine whether this trend held true for other aspects of the formulation, specifically assessing whether the incorporation of NaDES affected the integrity of the colloidal suspension.

The SLN-Blank-NaDES and SLN-AlClPc-NaDES formulations were subjected to colloidal stability analysis for 365 days under four storage conditions: ambient temperature (25 °C), refrigeration (4 °C), freezing (−20 °C), and heating (37 °C). Simple linear regression analysis was performed to assess the statistical significance of the stability across the different samples. The results are presented in Figure 6.

**Figure 6:**
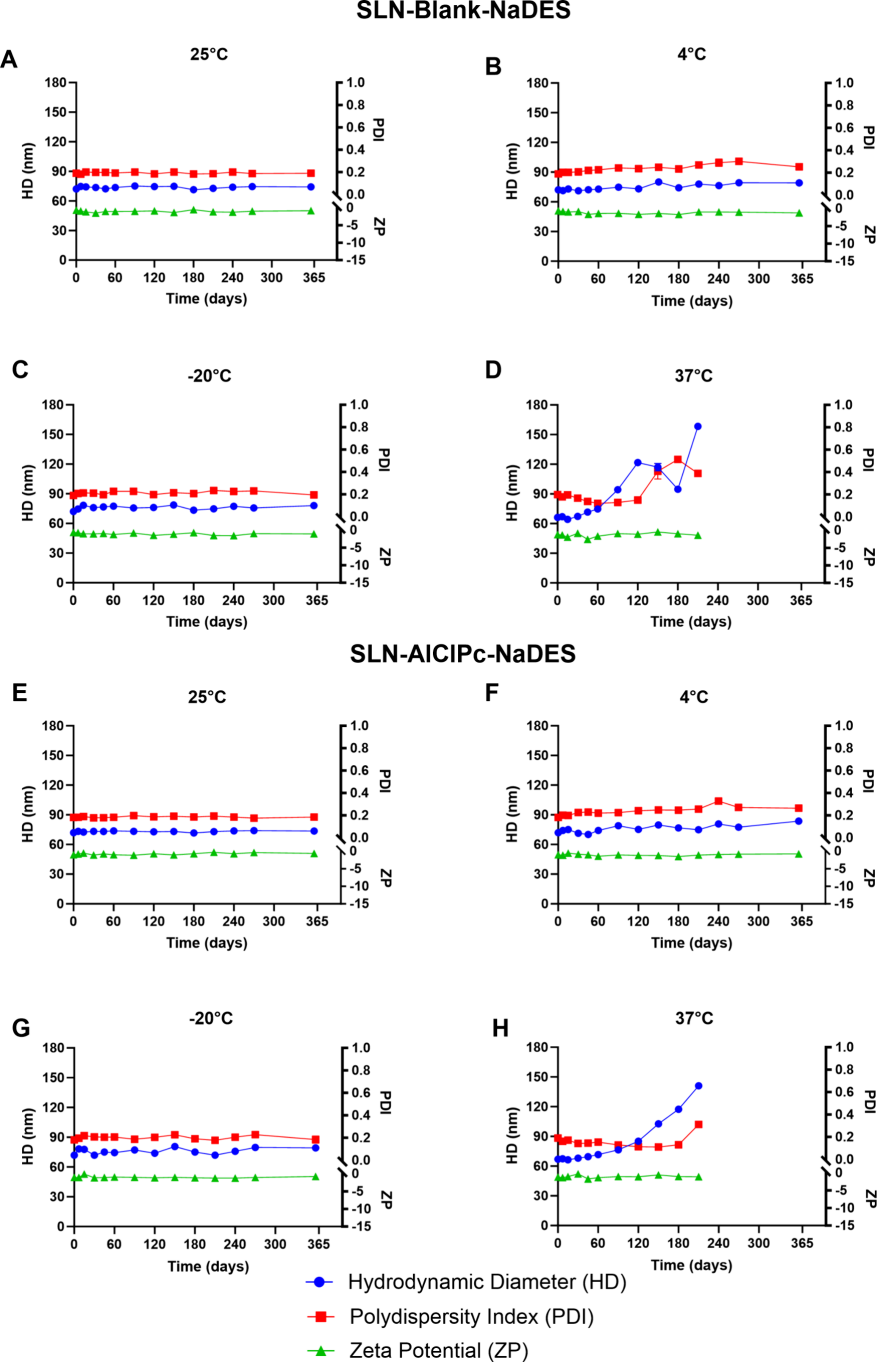
Colloidal stability of SLN-Blank-NaDES and SLN-AlClPc-NaDES. The graph shows the colloidal parameters of HD, PDI, and ZP over 365 days for the SLN-Blank-NaDES and SLN-AlClPc-NaDES formulations containing 12.5% solvent under the following conditions: (A, E) ambient temperature at 25 °C, (B, F) refrigeration at 4 °C, (C, G) freezing at −20 °C, and (D, H) heating at 37 °C. Data represent the mean ± SEM of triplicate formulations.

Both formulations developed showed stability under ambient temperature, refrigeration, and freezing conditions throughout the entire period, with no significant changes in any of the three parameters analyzed (HD, PDI, and ZP), i.e., *p* > 0.05. The incorporation of NaDES can induce changes in the interfacial properties between the lipid and aqueous phases. The hydrophilic components of the NaDES introduce hydrogen bonding interactions and increase the local polarity at the oil–water interface, which tends to reduce interfacial tension, thereby promoting formulation stabilization and enhancing photosensitizer retention within the SLNs.^52,76^

Our colloidal stability results provide supporting evidence for the compatibility between AlClPc and NaDES. The SLN-AlClPc-NaDES formulations remained monodisperse and stable throughout the entire experimental period, with no signs of photosensitizer recrystallization or precipitation, indicating that AlClPc remained solubilized and well-integrated within the SLN matrix. This behavior is consistent with literature reports on NaDES-based systems. For instance, Wikene et al.^77^ demonstrated that NaDES formulations containing citric acid preserve their hydrogen bonding networks even after extensive dilution.

However, under heating conditions at 37 °C, both SLN-Blank-NaDES and SLN-AlClPc-NaDES exhibited particle degradation, indicated by a significant increase in HD, especially after 90 days. The instability under heating is common in lipid nanoparticles and is justified by the high storage temperature, which promotes the degradation of the lipid (murumuru butter), whose melting point is 32.5 °C.^78^

The colloidal stability of the formulations under freezing conditions emphasizes the effective role of NaDES as a cryoprotectant in the formulation over extended periods while also highlighting the promising application of SLNs associated with a green solvent, which is a favorable aspect for their storage. Furthermore, the stability exhibited by the SLNs over long periods and under various conditions is a crucial factor, and in the industrial scaling process, it will certainly influence the reduction of storage and transportation costs, important factors for the commercial viability of a product.^79^

Upon analyzing the surface charge of the SLNs containing NaDES, as indicated by the ZP, a nearly neutral profile was observed, in contrast to the SLNs without NaDES developed by Mello et al.^27^ In acidic pH environments, such as that of the NaDES used in this study, ionizable compounds like citric acid tend to remain protonated, which may contribute to a reduction in surface charge.^80,81^

According to classical DLVO theory, high absolute values of ZP correspond to strong electrostatic double-layer repulsion, thereby promoting particle dispersion. Conversely, values approaching zero indicate diminished electrostatic repulsion. However, it is important to emphasize that this simplified interpretation does not necessarily imply instability, particularly in complex colloidal systems.^82^

In multiphasic formulations containing NaDESs, other mechanisms may overcome the reduced electrostatic repulsion. The presence of NaDES can promote various non-DLVO stabilization mechanisms that significantly contribute to the colloidal stability of nanoparticles. One such mechanism is steric stabilization, in which NaDESs molecules adsorb onto the surface of the nanoparticles, forming solvated protective layers that induce steric repulsion and prevent aggregation.^83^

Furthermore, the increased local viscosity caused by NaDESs significantly reduces the Brownian mobility of the nanoparticles, thereby attenuating the frequency of interparticle collisions and slowing down their translational motion, which enhances colloidal stability.^25^ Additionally, the formation of supramolecular networks via hydrogen bonding—a characteristic feature of NaDESs—leads to the development of a dynamic matrix surrounding the nanoparticles. This matrix entraps water molecules and organic components, generating a highly organized phase and inducing local rigidity, which further reduces effective collisions between particles.^84^

Thus, although the reduced ZP suggests a decrease in classical electrostatic repulsion, it should not be assumed that the nanoparticles will necessarily aggregate. This is supported by our findings, in which the nanoparticles remained stable over 365 days despite exhibiting a near-neutral surface charge, reinforcing the hypothesis of alternative stabilization mechanisms.

### Evaluation of the Role of NaDES in Enabling Drying Methodologies for SLN Concentration

Thus far, we have demonstrated the promising application of NaDES as a cryoprotectant for incorporation into the developed SLN formulations as well as the advantageous effects of its inclusion without compromising the formulation, which represents a key point of innovation in the present study.

Once established as cryoprotectants for SLNs, NaDES proved to be a potential tool for the standardization of previously presented freezing methodologies. These methodologies, in turn, play a crucial role in the implementation of concentration techniques that require prior cooling and freezing of the sample such as vacuum drying, rotary evaporation, and lyophilization. Therefore, we evaluated the contribution of the cryoprotective role of NaDES in these drying methodologies, aiming to enable protocols that were not yet feasible for the handling of SLNs.

#### Vacuum Drying and Centrifugation

The vacuum drying and centrifugation procedure resulted in a significant increase in the HD and PDI of all analyzed SLNs, particularly within the first three h of drying, during which a rising trend in these parameters was observed. The ZP also exhibited fluctuations throughout the entire period, although with smaller deviations (Figure 7).

**Figure 7:**
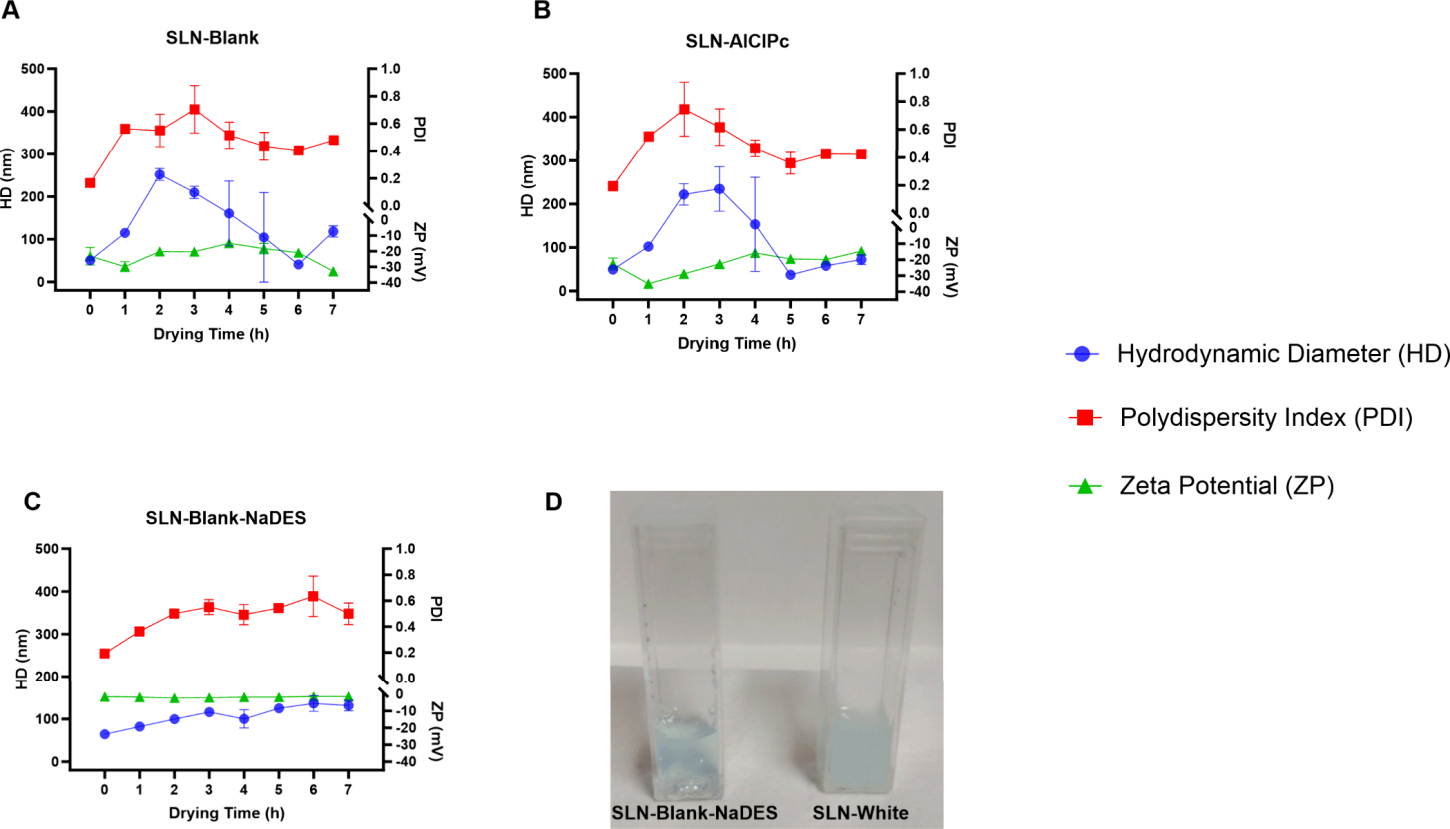
Colloidal parameters of the SLN-Blank, SLN-AlClPc, and SLN-Blank-NaDES formulations after vacuum drying and centrifugation. The graph depicts the colloidal parameters of HD, PDI, and ZP for SLN-Blank (A), SLN-AlClPc (B), and SLN-Blank-NaDES formulations containing 12.5% solvent (C) after 7 h of vacuum drying and centrifugation. Image D shows images of SLN-Blank-NaDES (left) and SLN-Blank (right) after 1 h of vacuum drying and centrifugation. Data are presented as the mean ± SEM of triplicate formulations.

The colloidal stability of SLN-Blank and SLN-AlClPc during the drying period showed similarity across all analyzed parameters (HD, PDI, and ZP) (Figure 7A,B), differing from that of the formulation containing NaDES. This similarity may be attributed to both formulations sharing the same lipid base, differing only in the incorporation of the photosensitizer.

It is noteworthy that the SLN-Blank-NaDES formulation exhibited distinct behavior compared with the others. Despite a significant increase in its colloidal parameters, with HD reaching 130 nm and PDI at 0.5, it showed smaller standard deviations (Figure 7C). This indicates that the addition of the solvent to the formulation may induce modifications that help reduce deviations, even though it does not preserve the colloidal properties.

The drying procedure also resulted in visually observable changes in the analyzed SLNs (Figure 7D), giving them a turbid appearance. A visual difference in the nanoparticles is evident as early as 1 h into the drying process. SLN-Blank-NaDES retained a more conserved macroscopic appearance compared with SLN-Blank, which exhibited greater turbidity and signs of degradation. However, this effect supports the previously presented results regarding the increase in the SLNs size, which was also evident macroscopically.

Based on the obtained results, the vacuum drying and centrifugation methodology was not considered suitable for concentrating the SLNs, and therefore, this assay was not conducted for the SLN-AlClPc-NaDES formulation.

#### Drying by Rotary Evaporation

The drying process of SLNs through rotary evaporation also increased the HD of all SLNs. However, with this methodology, the HD reached higher values, approaching 500 nm (Figure 8). The PDI values remained similar, with all formulations presenting a PDI of approximately 0.4 after rotary evaporation.

**Figure 8:**
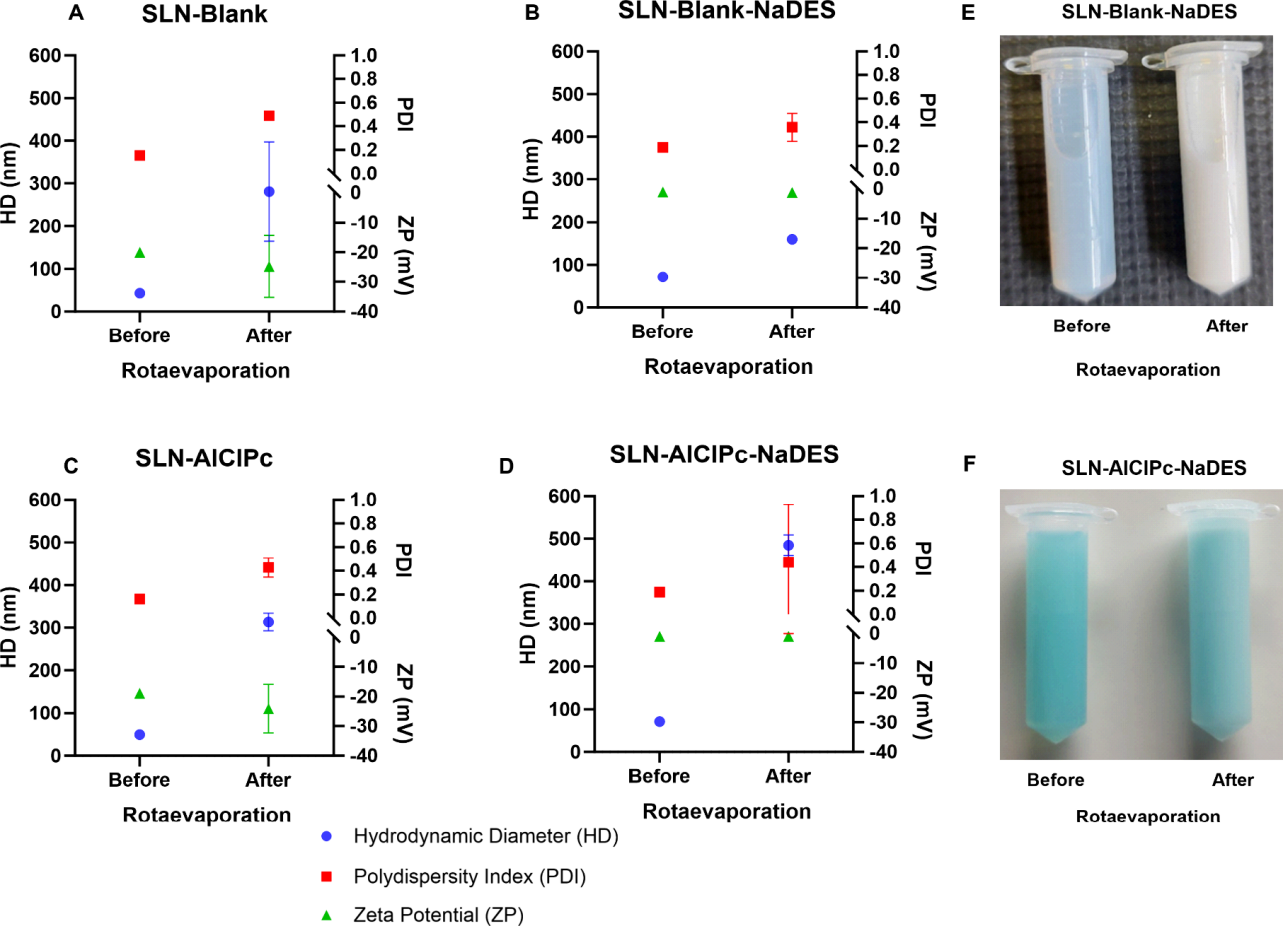
Colloidal parameters of SLN-Blank, SLN-Blank-NaDES, SLN-AlClPc, and SLN-AlClPc-NaDES formulations before and after rotary evaporation drying. The graphs show the colloidal parameters of HD, PDI, and ZP for SLN-Blank (A), SLN-Blank-NaDES containing 12.5% NaDES (B), SLN-AlClPc (C), and SLN-AlClPc-NaDES containing 12.5% NaDES (D) before and after a 5 h rotary evaporation drying procedure. Images of SLN-Blank-NaDES (E) and SLN-AlClPc-NaDES (F) before and after rotary evaporation drying are also presented. Data are presented as the mean ± SEM of triplicate formulations.

No significant differences were observed between the behavior of SLNs containing NaDES and those without the solvent in their formulation. Moreover, the rotary evaporation methodology resulted in samples with higher standard deviations, indicating low reproducibility and promoting particle degradation. Therefore, this methodology was not considered to be suitable for concentrating SLNs.

A more turbid appearance of the samples is observed after the drying process (Figure 8E,F). It is important to note that the SLNs subjected to rotary evaporation were resuspended to their original volume for macroscopic evaluation and comparison.

The study demonstrates that both vacuum drying with centrifugation and rotary evaporation did not result in viable samples as they caused severe alterations in the colloidal parameters of the nanoparticles, which could compromise their functionality and application. Furthermore, these methodologies were not found in the literature as nanoparticle concentration methods but are cited only as formulation techniques.^85,86^

#### Drying by Lyophilization

To promote particle concentration through lyophilization, the freezing tests previously conducted were used as a reference since this methodology requires prior freezing of the samples.

After the lyophilization process, an expressive increase in the HD of all SLNs was observed (Figure 9). For SLNs without NaDES in their formulation (Figure 9A,C), the process led to approximately a 2-fold increase in the HD of the particles as well as an increase in the PDI, which reached approximately 0.6. The ZP was not significantly altered.

**Figure 9:**
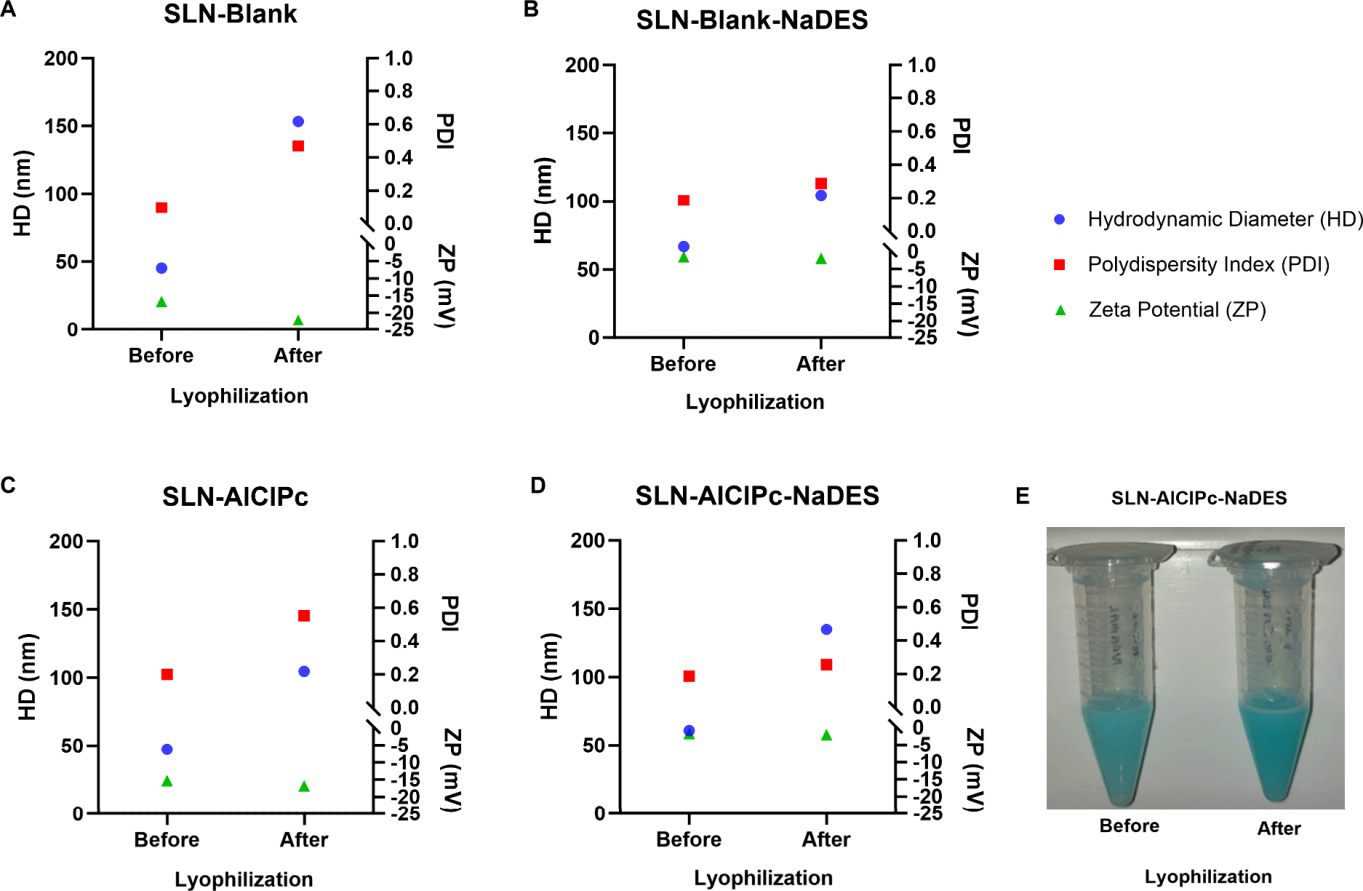
Colloidal parameters of SLN-Blank, SLN-AlClPc, SLN-Blank-NaDES, and SLN-AlClPc-NaDES formulations before and after lyophilization. The graphs show the colloidal parameters of the HD, PDI, and ZP for SLN-Blank (A), SLN-Blank-NaDES containing 12.5% NaDES (B), SLN-AlClPc (C), and SLN-AlClPc-NaDES containing 12.5% NaDES (D) before and after the lyophilization procedure (E). Data are presented as the mean ± SEM of triplicate formulations.

For SLN formulations containing NaDES ([Fig fig9]B,D), it is notable that the PDI remained almost unchanged, indicating that even after lyophilization, although the particles increased in size, they remained well dispersed, which is quite positive given the procedure stress to which they were subjected to.

Although cryoprotectants act by forming a protective layer around the particles, preventing aggregation during water removal, an increase in the HD often still occurs. This can be attributed to the strength of the bond formed between the cryoprotectants and water molecules, which can vary in strength. Similar results have been observed in other studies, where various cryoprotectants were used, and it was noted that the increase in particle size did not interfere with the other applications of SLNs.^18,19,87,88^

With regard to the visual and macroscopic appearance of the formulation after lyophilization and resuspension, it remained similar to that of the formulation not subjected to lyophilization, showing a translucent appearance and the same coloration (Figure 9E), with no signs of degradation despite undergoing different processes. This suggests a promising potential for the concentration methodology used. Given its success in effectively concentrating SLNs without significant alterations in their colloidal parameters, lyophilization was selected for further investigative assays

Morphological characterization by electron microscopy was performed on samples containing NaDES (before and after lyophilization). TEM results show that the nonlyophilized SLNs (SLN-Blank-NaDES and SLN-AlClPc-NaDES) exhibit spheroidal shapes and are monodisperse (Figure 10A,B).

**Figure 10:**
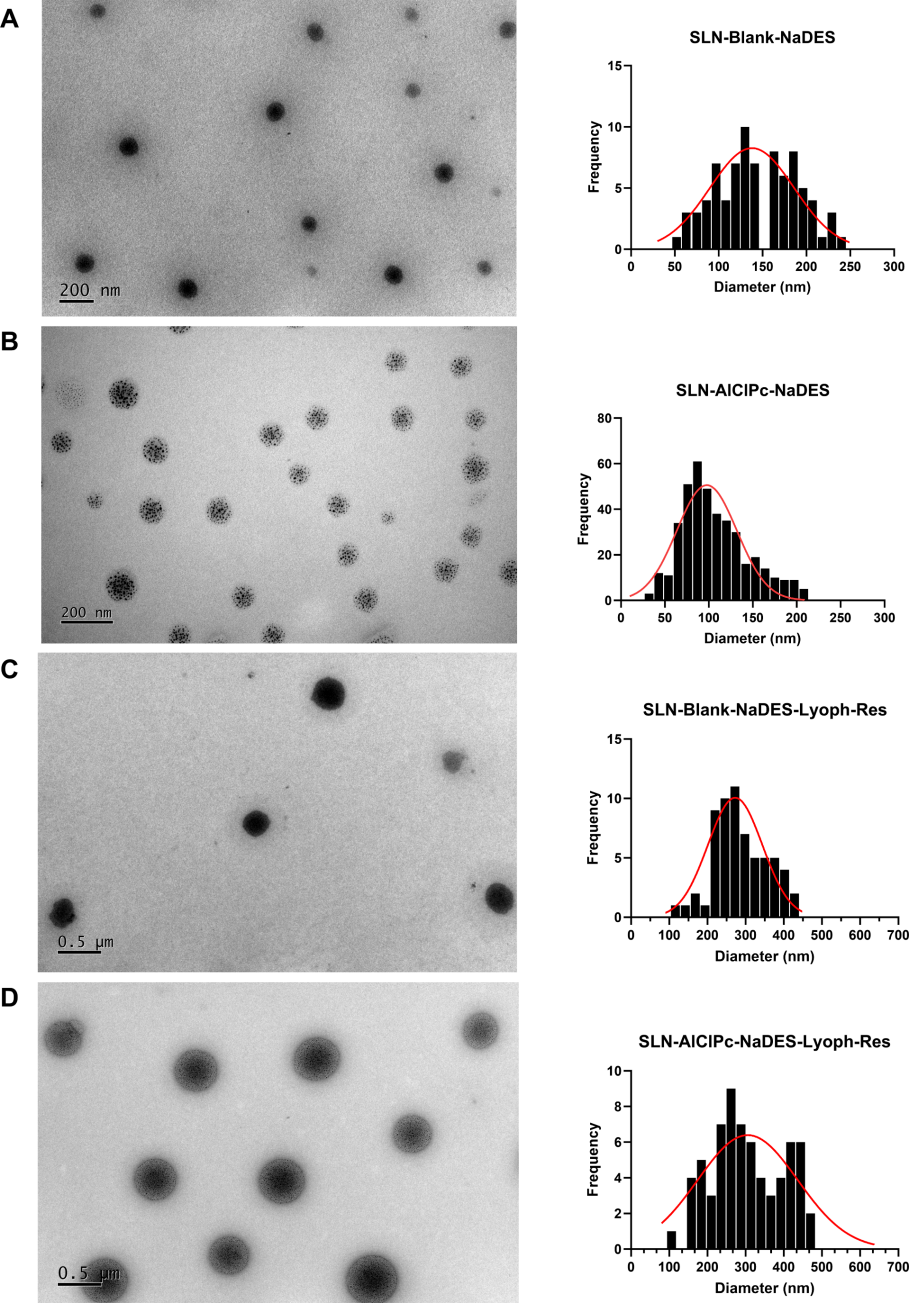
TEM images at 10,000× magnification of SLN-Blank-NaDES (A) and SLN-AlClPc-NaDES (B). TEM images at 5,000× magnification of SLN-Blank-NaDES-Lyoph-Res (C) and SLN-AlClPc-NaDES-Lyoph-Res (D). The corresponding histograms illustrating the particle size distribution are presented adjacent to each TEM.

Distinct morphological patterns were observed between the two formulations due to the incorporation of AlClPc, consistent with findings by Mello et al.^27^ in formulations without NaDES. In line with this pattern, SLN-Blank-NaDES seems more electron-dense but lose their polyhedral shape, probably due to the presence of the solvent in formulation. For SLN-AlClPc-NaDES, the structure remains very similar to that of SLN-AlClPc reported in the aforementioned study, with small darker regions, suggested to be AlClPc incorporated into the solid matrix of the nanoparticle.

For the particles subjected to lyophilization and subsequently resuspended (SLN-Blank-NaDES-Lyoph-Res and SLN-AlClPc-NaDES-Lyoph-Res), as shown in Figure 10C,D, the spheroidal shape and electrodensity profile were preserved, indicating that the concentration methodology did not induce significant structural changes. However, due to the lyophilization process, the particles exhibit an increase in size and greater polydispersity, consistent with the results observed through DLS analysis.

As observed in the micrographs obtained by scanning electron microscopy (SEM) in Figure 11, SLN-Blank-NaDES-Lyoph-Res and SLN-AlClPc-NaDES-Lyoph-Res exhibit a spherical shape and smooth surfaces. However, due to the lyophilization process, the particles present an increase and variation in size, leading to higher polydispersity. These findings corroborate with the results obtained from TEM.

**Figure 11:**
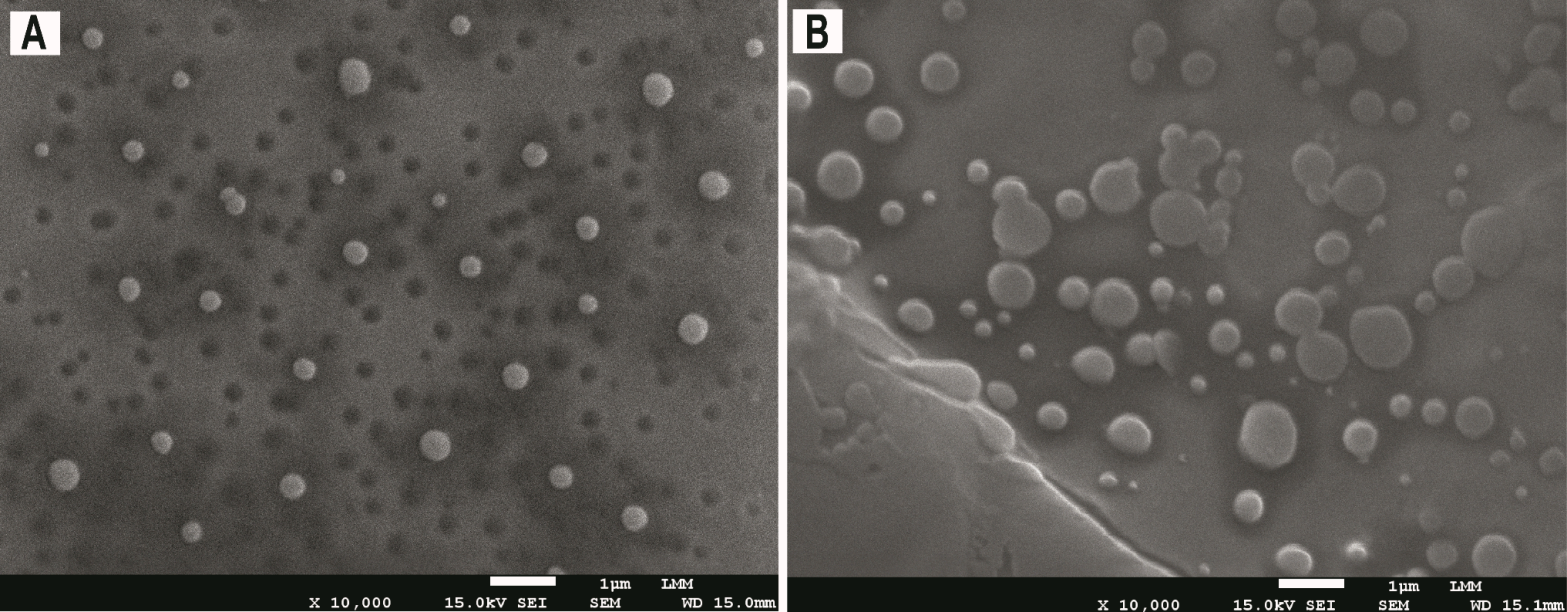
SEM images at 10,000× magnification of SLN-Blank-NaDES-Lyoph-Res (A) and SLN-AlClPc-NaDES-Lyoph-Res (B).

It was possible to observe the characteristic shape of the SLNs. However, the electron microscopy techniques did not allow visualization of the NaDES organization within the particle structure. Nonetheless, considering the characteristics of this solvent and the formulation, it is plausible that, being an amphiphilic solvent that can, in some cases, replace water molecule interactions, it may be present in the colloidal suspension with water, interacting with the nanoparticle surface. However, since this is a novel association, further experiments are necessary to better understand how this solvent is incorporated within the SLNs structure.

To assess the chemical and compositional modifications induced by the incorporation of NaDES, the lyophilization process, and the resuspension of the resulting nanocarriers (with and without AlClPc), the formulations were also analyzed by using FTIR spectroscopy. Figure 12 illustrates the obtained spectra and provides support for addressing the aforementioned points.

**Figure 12:**
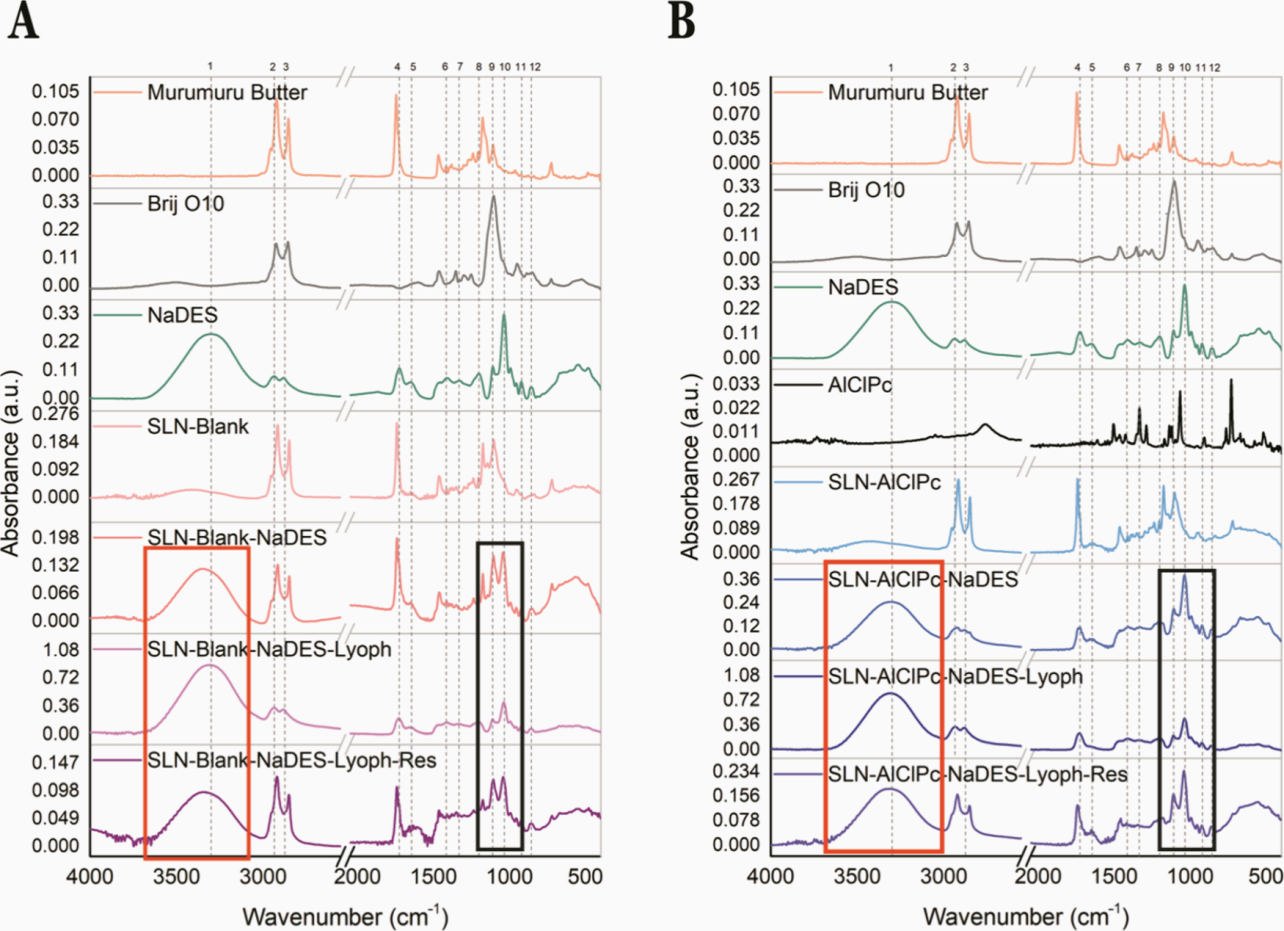
FTIR Spectroscopy of SLNs and their individual components. (A) Spectra of murumuru butter, Brij O10, NaDES, SLN-Blank, SLN-Blank-NaDES, SLN-Blank-NaDES-Lyoph (lyophilized), and SLN-Blank-NaDES-Lyoph-Res (lyophilized and resuspended). (B) Spectra of murumuru butter, Brij O10, NaDES, SLN-AlClPc, SLN-AlClPc-NaDES, SLN-AlClPc-NaDES-Lyoph (lyophilized), and SLN-AlClPc-NaDES-Lyoph-Res (lyophilized and resuspended). Red boxes highlight the increased intensity in the OH stretching region (∼3321 cm^–1^), observed in formulations containing NaDES. Black boxes indicate spectral changes associated with the lyophilization process, particularly in the δ­(OH) region, near 1000 cm^–1^.

When comparing the spectral profiles of formulations without AlClPc (Figure 12A) and with AlClPc (Figure 12B), the predominance of the lipid profile characteristic of all SLNs is observed, evidenced by the presence of murumuru butter, with bands at 2921 (2), 2850 (3), and 1741 cm^–1^ (4), and Brij O10, a surfactant derived from vegetable fatty ethers. Additionally, the incorporation of NaDES induces significant changes in absorption intensities within the OH band region (3321 cm^–1^ (1)), distinguishing NaDES containing formulations by the ν_as_(C–C–O) band (10) at 1035 cm^–1^, a characteristic feature of this solvent. These chemical modifications in the nanocarriers suggest that the incorporation of NaDES was successful, aligning with the research objective, particularly considering the partial substitution of water with this organic solvent.

Regarding the lyophilization process, SLN-Blank-NaDES were compared with SLN-Blank-NaDES-Lyoph (lyophilized) and SLN-AlClPc-NaDES with SLN-AlClPc-NaDES-Lyoph (lyophilized). The spectral profile of the nanocarriers, after lyophilization, undergoes significant alterations due to the absence of water in the sample, inducing variations in the chemical bonds/groups observed, which are notably distinct from the previous stage (SLN-Blank-NaDES and SLN-AlClPc-NaDES), in the region between 1830 and 400 cm^–1^. However, upon resuspension in an aqueous medium, the formulations acquire spectral profiles resembling those of the SLNs not subjected to the lyophilization process (SLN-Blank-NaDES and SLN-AlClPc-NaDES). This is observed in the samples SLN-Blank-NaDES-Lyoph-Res and SLN-AlClPc-NaDES-Lyoph-Res (lyophilized and resuspended), which display a mixed spectral profile, with characteristic peaks associated with both NaDES and the lipidic part, notably ν_as_ (CH_2_) (2) and ν_s_ (CH_2_) (3), ν­(C=O) (4), and δ­(OH) (9) groups.

This similarity suggests that the nanocarrier concentration methodology used preserves the formulations, as emphasized by the functional groups and spectral profiles observed. Thus, it can be inferred that even after the lyophilization process, the evaluated formulations maintained their integrity, presenting no significant signs of degradation, which underscores the potential of lyophilization as an effective methodology for the concentration of SLNs containing NaDES.

Electron microscopy allowed the observation of the typical morphology and structural features of SLNs, with denser lipid cores and the encapsulated active compound dispersed within this core, as expected for this class of nanocarriers. Given the innovative nature of this study, particularly the incorporation of NaDES into the formulations, it would also be of interest to understand how this solvent is organized within the SLNs structure. However, neither the electron microscopy techniques employed nor the FTIR analysis enabled visualization of the NaDES organization within the particles.

Nevertheless, considering the physicochemical characteristics of both the solvent and the formulation, it is reasonable to hypothesize that the NaDES, due to its amphiphilic nature and, in some cases, its ability to substitute for water molecules in hydrogen bonding networks, may remain in suspension alongside water, interacting at the nanoparticle surface.^89^ Similarly, just as the surfactant layer surrounding the particles or the aqueous dispersion medium cannot be visualized by transmission electron microscopy (TEM)—owing to sample preparation limitations that involve particle drying—the structural organization of the NaDES within the nanoparticle could also not be detected using this technique.

Despite this, considering the physicochemical attributes of the NaDES-based system and its interaction with both the lipid interface and the aqueous medium of the SLNs, it can be inferred that, given the hydrophobic nature of the SLN core formed by murumuru butter, the lipid matrix does not significantly contribute to the solubilization of the hydrophilic NaDES components. Instead, the interactions of this solvent are expected to be more pronounced at the interfacial region between the lipid and the aqueous phase, where the surfactant organizes the particle surface. In this regard, Brij O10, which is composed of polyoxyethylene hydrophilic chains, provides an interfacial region capable of establishing hydrogen bonds and electrostatic interactions with small polar molecules.^90^

In particular, glycerol, due to its high polarity and strong ability to form hydrogen bonds conferred by its hydroxyl groups, is more likely to remain in the aqueous phase, although it may also engage in interactions with the nanoparticle interface.^91^ By contrast, citric acid, as a weak organic acid with a variable ionization profile that depends on the medium, may exhibit interactions that vary according to its ionization state, which may allow its association either with the aqueous phase or with the nanoparticle interface involving the surfactant.^92^

Finally, choline chloride, a charged quaternary ammonium salt, is prone to strong ionic interactions that may favor its localization near charged or polar interfaces.^93^ This propensity further highlights the distinct affinities of each NaDES component toward different regions of the SLN system, thereby suggesting differentiated modes of interaction that may influence their overall performance.

Together, these findings complement one another and offer a more comprehensive examination of the role of NaDES in SLNs formulations, spanning both macroscopic characteristics and its chemical composition. This contributes to a deeper understanding of how this solvent can optimize the functionality of SLNs, thereby advancing their potential applications and facilitating their integration into industrial-scale processes.

Due to the complexity of the system under study, composed of multiple components that collectively contribute to the observed cryoprotective effect and are thoroughly discussed throughout the present work in association with SLN formulations, investigating the individual role of each component is of significant relevance. Such an analysis could provide a deeper understanding of the underlying molecular mechanisms of cryoprotection and help guide the development of optimized formulations, representing an important aspect to be explored in future studies.

Also, given the innovative nature of this association, further experimental studies and advanced characterization techniques are required to deepen our understanding of how NaDES components organize within the SLN structure. Techniques such as nuclear magnetic resonance (NMR), correlation spectroscopy, and molecular dynamics simulations are particularly relevant for elucidating the spatial distribution of the system’s constituents. Advancing this understanding represents a key priority and a natural perspective for the continuation of the present work.

### SLNs and NaDES: Unlocking Viability and a New Horizon for Scale-Up

Based on the results obtained in this study, combined with the expanding scientific evidence in the literature, the integration of nanocarrier-based delivery systems with NaDESs appears to be consistent with both laboratory-scale feasibility and scalability requirements.

SLNs have attracted increasing industrial interest due to their ease of preparation, physicochemical stability, and scalability potential, features that make them particularly promising for large-scale production. Simple SLNs formulation methodologies, such as high-pressure homogenization (HPH), as well as technologies like supercritical fluid processing or spray-drying, also enable the continuous production of SLNs at the industrial level, allowing for the processing of ton-scale quantities of material.^94^

Our study presents an even more scalable alternative: an adaptation of the PIT method developed by our research group, which is already protected by patents focused on the upscaling of these lipid-based nanosystems. This approach employs low-energy methods and natural, biocompatible compounds, such as murumuru butter.

In comparison with conventional solvent-based methods, such as solvent diffusion microemulsion or nanoprecipitation using volatile organic solvents, the use of NaDESs in SLN formulation offers a simpler and more sustainable alternative.^95^ As nonvolatile and ready-to-use solvents, NaDESs eliminate the need for solvent removal steps, thereby reducing processing costs and energy consumption.^20^ Furthermore, production methods employing NaDESs tend to be reproducible and scalable, avoiding the use of toxic solvents and resulting in formulations with good stability and uniform size distribution, attributes highly desirable for industrial applications.^96^

All constituents of the NaDES employed in the present study (choline chloride:glycerol:citric acid) are of natural origin or safe synthetic sources, previously used in food and pharmaceutical products. They are readily available, exhibit low toxicity, and are noted for their low cost and ease of large-scale synthesis.^97–99^

There are already indications of industrial interest in systems based on NaDESs, which are particularly driven by the growing demand for green processes. Studies such as that by Schuh et al.^62^ have demonstrated strategies aimed at scalability, highlighting this as a strong trend. In their work, the authors present the NLC-NaDES platform as robust for various applications, capable of achieving efficacy comparable to or exceeding that of conventional methods while significantly reducing the use of toxic solvents and water during production. Other studies in green chemistry highlight NaDESs as an environmentally friendly alternative with broad industrial applicability.^100^

Within this context, the combination of NaDESs with lipid nanoparticles aligns with the principles of green chemistry and Industry 4.0, providing industrial flexibility, reduction of toxic byproducts, and the utilization of low-cost natural feedstocks.^101^ Such technical and environmental evidence supports the economically sustainable characteristics for the potential industrial upscaling of SLN systems associated with NaDESs (such as choline chloride:glycerol:citric acid).

Although proposed as an initial strategy toward scaling, this approach contributes to consolidating a technical-scientific foundation that underpins the advancement of these platforms toward sustainable innovation and large-scale technological development. It is important to emphasize, however, that the present work remains at a preliminary stage, and the transition to an industrial setting has not yet been implemented. Therefore, the findings and discussions presented should be interpreted from a prospective standpoint as an early step toward future scalability.

## Conclusions

This study introduced an innovative approach to the production and preservation of SLNs by incorporating an NaDES as a sustainable cryoprotectant. Our findings demonstrated that this technology maintains colloidal stability during freezing and enables efficient concentration via lyophilization without compromising the physicochemical and structural properties of the nanoparticles. Additionally, the use of NaDES showed the potential to improve the solubilization of AlClPc, suggesting a possible expansion of its pharmaceutical applications.

Notably, this research constitutes the first empirical validation of NaDESs as cryoprotectants for SLNs, an innovation secured under patent registration (registration number: BR 10 2025 002622 8), and represents a significant step toward the industrial applicability of nanoparticle-based technologies. Moreover, the partial replacement of water with NaDES in SLN formulations emerges as a potentially effective strategy for developing more sustainable and efficient preservation protocols. This approach may, in the future, support the scalability of such formulations by reducing the reliance on toxic solvents and aligning with greener production practices.

It is important to note that this investigation focused on a single NaDES formulation, chosen based on its biocompatibility, affordability, and ease of preparation. Given that distinct combinations of hydrogen bond donors and acceptors can exhibit diverse physicochemical behaviors—and consequently, varying performance as cryoprotectants—future studies are needed to explore and compare alternative NaDESs formulations, particularly in terms of preservation efficacy, toxicity, and nanoparticle stability.

The proposed formulation demonstrates potential for industrial scalability due to the simplicity, reproducibility, and cost-efficiency of the processes involved, although validation under practical production conditions and more comprehensive assessments are required. Although the components are predominantly derived from potential biodegradability sources, the generation of nonrecyclable waste remains a concern for large-scale applications and further studies, including biodegradability assessments and extensive ecotoxicological evaluations.

Therefore, the presented findings offer a foundation for addressing long-standing limitations in SLN preservation and open new avenues for the development of lipid-based nanocarriers, positioning them as a promising alternative for biomedical and pharmaceutical applications.

## Supplementary Material



## Notes

Figure S1: FTIR spectra for all NaDES compounds; Figure S2: Colloidal parameters of SLN-Glycerol containing 2.5, 5, 7.5, 10 and 20% of glycerol before (A) and after freezing at −20 °C (B) (PDF)
