# Supplementary material for: Incorporation of a Natural Deep Eutectic Solvent-Based System as a Cryoprotectant in Solid Lipid Nanoparticles: Advancing toward Industrial Scalability
Source: ACS Nanosci Au. 2025 Oct 1;5(6):504–26. doi: 10.1021/acsnanoscienceau.5c00097 (PMC12715630; doi:10.1021/acsnanoscienceau.5c00097)
Supplement: Supplementary file 1 [file ng5c00097_si_001.pdf]

Supporting Information

Incorporation of a Natural Deep Eutectic Solvent-Based System as Cryoprotectant in Solid Lipid Nanoparticles: Advancing Towards Industrial Scalability

Isadora Florêncio [a,b], Marina M. Simões [a], Karen L. R. Paiva [a], Luane de Almeida Salgado [a], Ariane P. Silveira [a,c], Tathyana B. Piau [d], Cesar K. Grisolia [d], Victor Carlos Mello [a,c], Sônia N. Báo [a]*

[a] *Laboratory of Microscopy and Microanalysis, Department of Cell Biology, Institute of Biological Sciences, University of Brasília, Brasília 70910-900, DF, Brazil;*

[b] *Laboratory of Nanobiotechnology, Department of Genetics and Morphology, Institute of Biological Sciences, University of Brasilia, Brasília 70910-900, DF, Brazil;*

[c] *Cooil Cosmetics, Brasília 72622-401, DF, Brazil;*

[d] *Laboratory of Genetic Toxicology, Department of Genetics and Morphology, Institute of Biological Sciences, University of Brasília, Brasília 70910-900, DF, Brazil.*

**Email: snbao@unb.br*

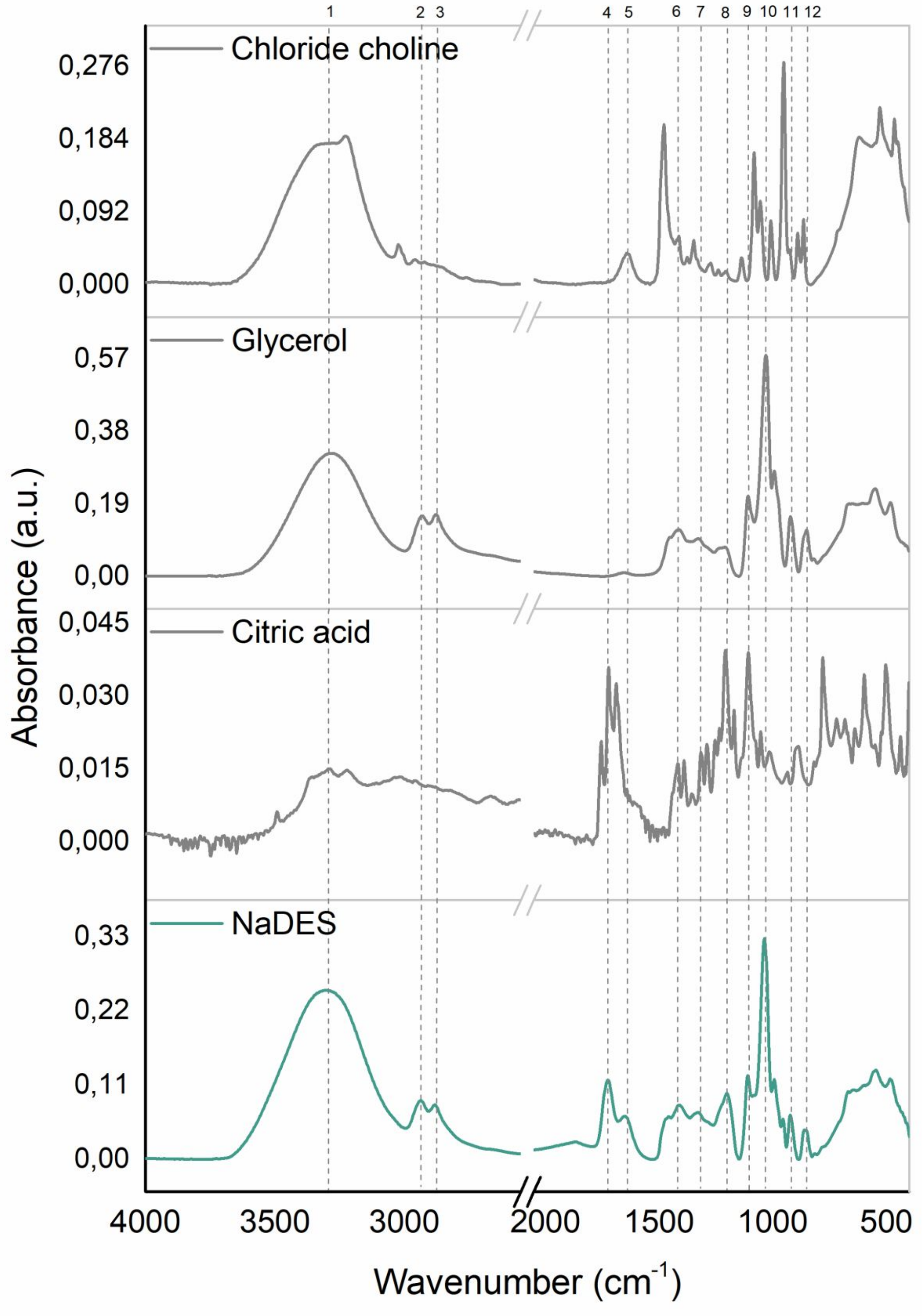

Figure S1: FTIR spectra for all NaDES compounds.

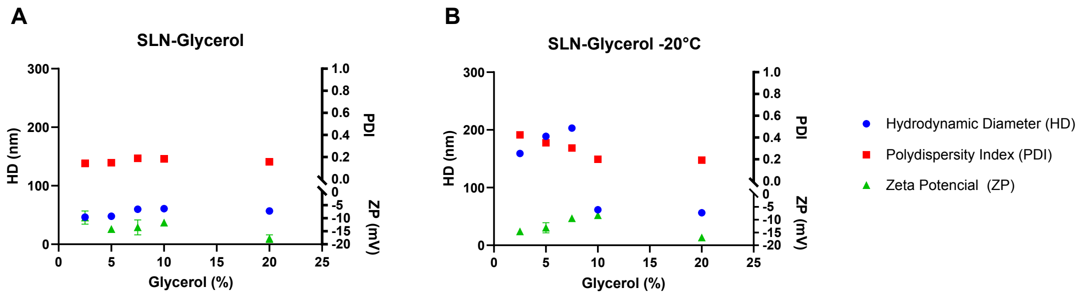

Figure S2: Colloidal parameters of SLN-Glycerol containing 2.5%, 5%, 7.5%, 10% and 20% of glycerol before **(A)** and after freezing at -20˚C **(B)**.
